# Exercise Training Induces a Shift in Extracellular Redox Status with Alterations in the Pulmonary and Systemic Redox Landscape in Asthma

**DOI:** 10.3390/antiox10121926

**Published:** 2021-11-30

**Authors:** Anna Freeman, Doriana Cellura, Magdalena Minnion, Bernadette O. Fernandez, Cosma Mirella Spalluto, Denny Levett, Andrew Bates, Timothy Wallis, Alastair Watson, Sandy Jack, Karl J. Staples, Michael P. W. Grocott, Martin Feelisch, Tom M. A. Wilkinson

**Affiliations:** 1 Clinical and Experimental Sciences and Southampton Centre for Biomedical Research, Faculty of Medicine, University of Southampton, Southampton SO16 6YD, UK; dc1v08@soton.ac.uk (D.C.); M.Minnion@soton.ac.uk (M.M.); B.Fernandez@soton.ac.uk (B.O.F.); C.Spalluto@soton.ac.uk (C.M.S.); D.Levett@soton.ac.uk (D.L.); Andrew.Bates2@uhs.nhs.uk (A.B.); Timothy.Wallis@soton.ac.uk (T.W.); A.S.Watson@soton.ac.uk (A.W.); S.Jack@soton.ac.uk (S.J.); K.Staples@soton.ac.uk (K.J.S.); mike.grocott@soton.ac.uk (M.P.W.G.); M.Feelisch@soton.ac.uk (M.F.); T.Wilkinson@soton.ac.uk (T.M.A.W.); 2 NIHR Southampton Biomedical Research Centre, University Hospitals Southampton NHS Foundation Trust, Tremona Road, Southampton SO16 6YD, UK

**Keywords:** asthma, exercise, oxidative stress, reactive species interactome, inflammation

## Abstract

Redox dysregulation and oxidative stress have been implicated in asthma pathogenesis. Exercise interventions improve symptoms and reduce inflammation in asthma patients, but the underlying mechanisms remain unclear. We hypothesized that a personalised exercise intervention would improve asthma control by reducing lung inflammation through modulation of local and systemic reactive species interactions, thereby increasing antioxidant capacity. We combined deep redox metabolomic profiling with clinical assessment in an exploratory cohort of six female patients with symptomatic asthma and studied their responses to a metabolically targeted exercise intervention over 12 weeks. Plasma antioxidant capacity and circulating nitrite levels increased following the intervention (*p* = 0.028) and lowered the ratio of reduced to oxidised glutathione (*p* = 0.029); this was accompanied by improvements in physical fitness (*p* = 0.046), symptoms scores (*p* = 0.020), quality of life (*p* = 0.046), lung function (*p* = 0.028), airway hyperreactivity (*p* = 0.043), and eosinophilic inflammation (*p* = 0.007). Increased physical fitness correlated with improved plasma antioxidant capacity (*p* = 0.019), peak oxygen uptake and nitrite changes (*p* = 0.005), the latter also associated with reductions in peripheral blood eosinophil counts (*p* = 0.038). Thus, increases in “redox resilience” may underpin the clinical benefits of exercise in asthma. An improved understanding of exercise-induced alterations in redox regulation offers opportunities for greater treatment personalisation and identification of new treatment targets.

## 1. Introduction

Exercise was long known to be beneficial for health and is increasingly recognised in the prevention and modification of disease processes [1,2]. Despite appreciation of its widespread benefits, the mechanisms through which these effects are conferred are not well understood. An acute bout of exercise induces oxidative stress in skeletal muscle and triggers an orchestrated molecular response at the whole-body level with thousands of changes in the metabolomic, proteomic and lipidomic profile [3,4]. Much less is known about systems-wide effects of repeated exercise, but improved redox regulation was proposed as a key mechanism through which physical exercise exerts its systemic effects [5]. The observation that antioxidant supplements interfere with the health-promoting effects of exercise [6] suggests improvements are mediated via modulation of redox signalling, possibly accompanied by an enhanced antioxidant defence capacity.

Redox dysregulation and oxidative stress were implicated in the pathogenesis of asthma [7,8] and may be a mechanism common across asthma phenotypes and endotypes. Asthmatic lung tissue is exposed to a range of exogenous and endogenous reactive oxygen (ROS) and nitrogen species (RNS) at considerably higher amounts than healthy lungs due to chronic inflammation. Exogenous sources include air pollutants, particulates, and cigarette smoke, with endogenous ROS produced as by-products of mitochondrial respiration, inflammatory cell responses to allergens and microbial infections [8,9]. Allergen challenge [10] and acute exacerbations can further increase ROS production in the lungs of asthmatics [11]. During acute asthma exacerbations, overall antioxidant capacity of plasma is reduced [11], indicating that local inflammatory events can affect systemic redox status by depleting extracellular buffering capability. Thiol redox disturbances were documented in children with severe asthma [12], with impact on glucocorticoid responsiveness associated with increased levels of oxidation [13]. More recently, endoplasmic reticulum stress, mitochondrial dysfunction, NADPH oxidases, and glutathionylation chemistry were linked to proinflammatory signalling in chronic lung diseases [14,15] and hydrogen sulfide was proposed as a novel biomarker of asthma [16]. Together, these findings suggest that aberrant redox regulation plays a central role in the pathogenesis of asthma.

Exercise acts as an oxidative stressor, triggering redox-sensitive signalling responses [17], with the redox responses to exercise exhibiting wide variability between individuals [18]. Regular exercise was proposed to increase the antioxidant defences of the body and provide an overall increase in the ability to counteract oxidative stress [19]. In agreement with this notion, exercise interventions in asthma were shown to improve symptoms and increase quality of life; a recent review concluded that exercise intervention may reduce airway inflammation in patients with asthma [20,21]. Two conceptual frameworks, the ‘Reactive Species Interactome (RSI)’ and the ‘Redox Interactome’, were introduced to help us understand how cells, organs, and whole organisms sense and adapt to changes in environmental and metabolic challenges [22,23]. The RSI framework also highlighted the limitations of biomarker research in as much as all reactive species interact with one another, forming a singular complex reaction network [22,24,25]. At the same time, this framework highlighted opportunities for its assessment by considering the extracellular fluid as a “communication highway” through which the lung is connected to all other metabolically active organs [26]. We sought to use these new concepts to explore how repeated exercise-induced oxidative stress modulates systemic redox regulation in asthma. Specifically, we hypothesized that a well-controlled exercise intervention would translate into a reduction in inflammation and an improvement in overall antioxidant capacity as a result of the whole-body response to repeated bouts of oxidative stress. To address this, we performed an exploratory prospective clinical study in a small number of patients with symptomatic asthma to determine the impact of personalised and metabolically controlled interval exercise training on the primary endpoint of asthma-related clinical outcomes, whilst in parallel also investigating their extracellular redox status by quantifying stable readouts of the whole-body RSI status.

## 2. Materials and Methods

### 2.1. Subjects

This study was approved by South Central-Hampshire B Research Ethics Committee 17/SC/0256, and all participants provided informed consent. We recruited patients between 18–80 years of age with a diagnosis of asthma and evidence of airway hyper-responsiveness or diurnal variation (of greater than 8% on peak flow diary or >12% and 200 mL on lung function testing). Participants were identified as symptomatic at recruitment with an Asthma Control Questionnaire (ACQ) score of >1.5 [27], and physically inactive as defined by the American College of Sport’s Medicines guidelines of <60 min of structured or planned physical activity per week. Current (<6 months cessation) or ex-smokers with a >10 pack year history and patients with an exacerbation within the last 4 weeks requiring treatment with either antibiotics or steroids were excluded. Those with a change in asthma medications during the study period, another clinically significant respiratory or inflammatory disease, a contraindication to cardiopulmonary exercise testing (CPET), or females who were pregnant or breastfeeding, were also excluded.

### 2.2. Exercise Intervention and Testing

Patients undertook a symptom-limited, incremental, cardiopulmonary exercise test (CPET), performed to a local standard operating protocol (SOP), to determine oxygen uptake at anaerobic threshold (AT; determined using the V-slope method by 2 independent reviewers blinded to patient history and intervention) and peak exercise (VO2peak) capacity [28]. Workloads at anaerobic threshold and peak exercise were utilised to prescribe an interval exercise intervention that delivers equivalent metabolic and oxidant stimuli [2,29]. Participants undertook a thrice weekly, 30 min, supervised, in-hospital interval exercise training programme on an electromagnetically braked cycle ergometer (Optibike Ergoline GmbH, Germany) for a period of 12 weeks. Training intensities were determined by measured work rates at AT and peak exercise, as determined by CPET at baseline, 3, 6 and 12 weeks. Training workloads were modified to account for physiological adaptation to the training programme. The training sessions comprised 3 min at moderate intensity (workload of 80% of AT), followed by 2 min at severe intensity (at a work rate halfway between AT and VO2peak), repeated for 4 cycles in week 1 and 6 cycles for the remainder of the programme, with an additional 5 min warm-up and cool-down time [2]. Two participants additionally undertook resistance exercise training performed at 60% of the 1 repetition maximum. CPET data were reported blind by two independent assessors with reporting experience to the level of accreditation requirement with the Peri-Operative Exercise Testing and Training Society. Mean values were used. Differences of >5% were resolved by a third expert practitioner.

### 2.3. Sample Collection and Processing

Sampling was undertaken at baseline, 3 weeks (limited sampling), 6 weeks and within 5–10 days of completion of the exercise intervention.

Venous blood was collected in serum tubes and EDTA vacutainers before acute exercise challenge (CPET) for analysis of inflammation markers, nitric oxide (NO) metabolites, thiol metabolome status, and measurement of extracellular antioxidant capacity. Additional samples were taken immediately post CPET for NO metabolites, thiol metabolome and antioxidant capacity analysis. Serum was collected by centrifugation of clotted blood after 1 h at room temperature. EDTA blood was subjected to immediate centrifugation at 1200× *g* for 20 min to obtain plasma, pipetted in 1 mL aliquots into cryovials (Nunc), snap-frozen in liquid nitrogen and stored at −80 °C for later biochemical analyses. Portable RTube^TM^ devices (Respiratory Research Inc., Austin, TX, USA) were used to collect exhaled breath condensate (EBC), and the procedure was conducted in accordance with the EBC collection rules published by the American Thoracic Society/European Respiratory Society (ATS/ERS) [30]. To reduce background contaminant levels of nitrite and nitrate, all glassware, collection tubes and pipettes were rinsed with ultrapure water and dried before use.

Systemic inflammation was quantified through full blood count (FBC) and C-reactive protein (CRP) determination, and via Luminex xMAP cytometric bead array assay of plasma interleukin (IL)-5, IL-6, IL-10 and IL-13, IL1-ra, CCL-11/eotaxin, tumour necrosis factor-alpha (TNFα) and interferon gamma (IFNγ) concentrations (Luminex 200^TM^, R&D Systems, Austin, TX, USA).

Sample aliquots allocated for NO metabolites and thiol metabolome analysis were pre-treated with N-ethylmaleimide (NEM) at time of collection, whereas those for other biomarkers were used immediately after thawing. Daily calibrations with authentic standards were performed for all biochemical assays; to keep processing times comparable, thawing and sample processing was conducted in batches of 5–10 at a time. All colorimetric assays were performed in 96-well plate format using a SpectraMax M5 microplate reader (Molecular Devices, San Jose, CA, USA).

Nitrate and nitrite concentrations were quantified using a dedicated high-performance liquid chromatography analysis system (ENO-20, Amuza Inc., San Diego, CA, USA), following sample deproteinization by methanol precipitation and centrifugation [31]. Total nitrosation products (RXNO) in EBC or NEM-pretreated plasma were quantified by gas-phase chemiluminescence detection (CLD 77am sp, EcoPhysics, Duernten, Switzerland) of bound NO, following removal of nitrite with acidified sulfanilamide and reduction of nitroso species by acidic triiodide, as described [32]. The concentrations of free sulfide and small aminothiols, including reduced and oxidized forms of cysteine, homocysteine and glutathione, were quantified in NEM pre-treated plasma using ultrahigh-pressure liquid chromatography-electrospray ionization-tandem mass spectrometry (UPLC-ESI-MS/MS); free and bound thiols were quantified before and after reduction of plasma aliquots by dithiothreitol, as described [33].

Total free thiol (TFT) concentrations in serum, which largely reflects the availability of a single free cysteine group (Cys-34) of albumin, were determined spectrophotometrically using Ellman’s reagent (5,5’-dithio-bis-2-nitrobenzoic acid; DTNB) and normalised to protein concentration, as described [34].

The ferric reducing ability of plasma (FRAP) was used as a measure of total antioxidant capacity [30]. This assay measures the reduction of ferric (Fe^3+^) to ferrous (Fe^2+^) ions by the formation of an intense, blue-coloured ferrous-tripyridyltriazine complex under acidic conditions; Fe^3+^ reacts with a variety of reducing compounds, including uric and ascorbic acid, but not thiols. A standard curve of known concentrations of ferrous ions was used to compare with absorbances of Fe^3+^-reacted plasma samples at 593 nm.

Whole-body lipid oxidation status was estimated in EDTA plasma samples using the thiobarbituric acid reactive substances (TBARS) assay, with known concentrations of the tetrabutylammonium salt of malondialdehyde (MDA) as reference [35,36]. MDA (and other reactive aldehydes generated by (per)oxidation of polyunsaturated fatty acids) reacts with thiobarbituric acid (TBA), at high temperature and acidic conditions, to form a pink 1:2 MDA-TBA adduct, with maximum absorbance at 532 nm.

Lung function testing including pre- and post-bronchodilator spirometry and fractional exhaled nitric oxide (FeNO) measurements were performed using a CLD 88 sp analyzer with Spiroware software package V3.2 (EcoMedics, Duernten, Switzerland). Asthma Control Questionnaire (ACQ) [27] and Asthma Quality of Life Questionnaire (AQLQ) [37] were also completed.

### 2.4. Outcome Measures

The primary clinical outcome was improvement in asthma symptoms as assessed by Asthma Control Questionnaire (ACQ) 6 score [27], with secondary clinical outcomes including asthma related quality of life, as assessed by the Asthma Quality of Life Questionnaire (AQLQ) [37]. Spirometry was performed according to standard protocols in accordance with ERS/ATS guidelines [38], as previously reported [39]. Arthrometric data were measured using the Seca mBCA (medical Body Composition Analyser) (Seca, Birmingham, UK) and airway inflammation was assessed using FeNO, using local SOPs, as previously detailed [39]. In addition, inflammatory status was assessed by measurement of nitroso product concentration in EBC. Systemic inflammation was quantified through FBC and CRP, and via inflammatory cytokine profile mapping while changes in downstream markers of redox regulation were assessed using locally developed and published techniques, as described above.

### 2.5. Data Analysis

This was an exploratory study, as there are few data in the literature describing deep phenotyping studies in asthma patients, specifically in response to exercise. It was therefore not possible to perform a single statistical assessment to ensure adequate power to detect differences in the redox metabolome following the intervention. However, our measurement methods were demonstrated to be robust, specific and sensitive [33]. A clinically significant improvement in ACQ score was previously defined as ≥−0.5 [40] and for the AQLQ as ≥0.5 [37]. For increase in oxygen uptake, a minimal clinically important difference (MCID) of ≥2 mL/kg/min is routinely used [41].

All data were treated as nonparametric, and data analysed on a per protocol basis. Significance was assumed if *p* < 0.05 using the Wilcoxon Signed-Rank Test or Friedman Test. Bivariate correlations between physical fitness, the redox metabolome and clinical markers of asthma were assessed using a Spearman’s test. An r value of >0.7 was considered a strong correlation, an r value of 0.4 to 0.7 was considered a moderate correlation. Repeated measures adjustments were not employed due to the small sample size and difficulty in accurately assessing data of this size for normal or non-normal distribution [42]. Calculations were performed using IBM SPSS 25 (IBM, Chicago, IL, USA) and figures were produced using GraphPad Prism 8 (GraphPad Software, San Diego, CA, USA).

## 3. Results

Twenty-four patients were enrolled in the study. Data are presented for the 6 participants who completed the exercise training intervention and attended all sampling visits. Adherence to planned training was good, with median adherence to training sessions of 86% (IQR 81.5–92.75%). Overall dropout rates were substantial due to the time burden of the in-hospital training format that was employed for safety and scientific rigour of this exploratory study; see Supplement (Figure S1).

### 3.1. Demographics and Physiological Data

Demographic data for the participants (*n* = 6) are listed in Table 1, with the patient group reflecting the wider asthma population with a high prevalence of atopy, raised peripheral blood eosinophil counts, and co-morbidities common in difficult asthma [43]. The group were in the overweight category for body mass index (BMI) at enrolment, and this did not significantly change following the exercise intervention (*p* = 0.17). Body composition, in terms of fat-free mass, visceral adipose tissue and skeletal muscle mass remained stable over the course of the intervention (Table 2). The group demonstrated an increase in physical fitness after the intervention, when assessed as oxygen uptake at AT (median change 1.65 mL/kg/min (IQR 0.66, 2.1, *p* = 0.046) and a clinically significant trend to increase in oxygen uptake at peak exercise (median change 3.75 mL/kg/min; IQR 0.48, 4.6, *p* = 0.058, MCID 2 mL/kg/min). Participants were able to increase their maximum workload significantly (median change 27 watts; IQR 17.3, 38.5) (Table 2).

### 3.2. Asthma-Related Clinical Outcomes

Asthma symptom scores showed clinically meaningful (MCID ≥ −0.5) and statistically significant improvements between baseline and week 12 (median change = −0.58; IQR −1.5, −0.9, *p* = 0.02). Asthma-related quality of life (QoL) improved between baseline and week 12 (median change 1.12; IQR 0.5, 1.9 *p* = 0.046), with an MCID of ≥0.5. When AQLQ domains were assessed independently there were improvements in the symptoms, emotional and environmental domains, but not the activity domain (Table 3). Pre-bronchodilator forced vital capacity (FVC) demonstrated a significant improvement between baseline and post intervention (*p* = 0.028), with a significant reduction in forced expiratory volume in 1 s % predicted (FEV1%) bronchodilator reversibility (*p* = 0.043), markers of lung function and airway responsiveness. There was no statistically significant change in FEV1 upon intervention but there was a clinically relevant improvement of 290 mL overall (see Supplementary Data; Table S1).

Airway inflammation, as assessed by FeNO levels, did not appear to change (Table 4). However, the concentrations of nitrite, nitrate and nitroso species in EBC displayed highly heterogenous fluctuations (see Supplementary Figures S2 and S3), suggestive of complex alterations in oxidative and nitrosative chemistry of NO within the alveolar fluid. Systemic inflammation reduced over the course of the intervention, as assessed in peripheral blood sampled before an acute exercise challenge: total white cell (*p* = 0.046), lymphocyte (*p* = 0.049) and eosinophil counts (*p* = 0.007) all demonstrated significant reductions (Table 4). Plasma CCL11/eotaxin, IL-5, TNFα and IFNγ levels were also reduced by the exercise intervention (*p* = 0.046) (Table 4).

### 3.3. The Integrated Adaptive Redox Response to Exercise in Patients with Asthma

**Figure 1:**
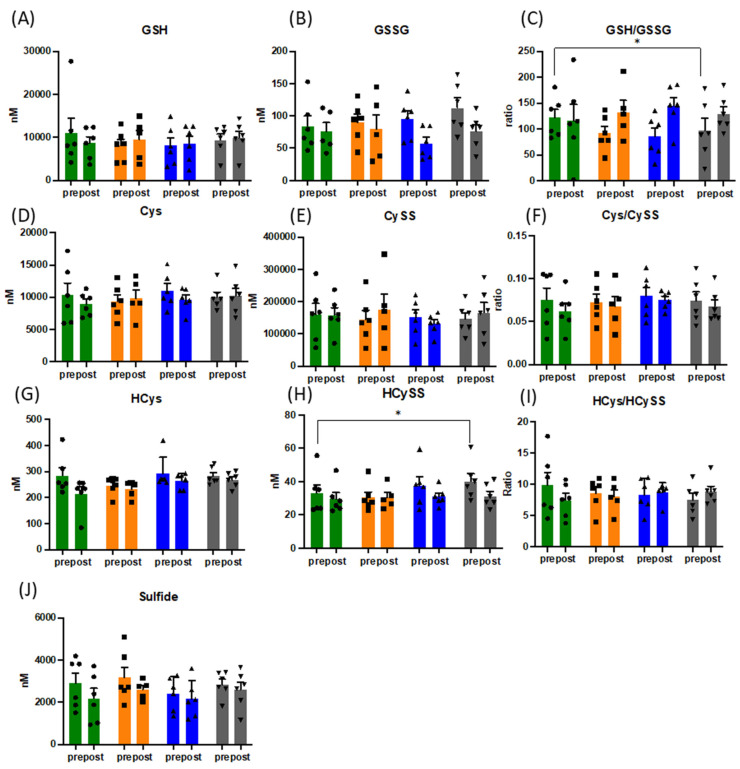
Changes in pattern of responses of the free thiol metabolome (**A**–**J**). Data presented for *n* = 6 before and after acute physiological challenge of a cardiopulmonary exercise test, presented as mean and SEM, with overlaid individual data (circles, squares, up- and downward triangles). Data presented in nM or concentration ratio. Green bars = baseline, orange bars = week 3, blue bars = week 6 and grey bars = week 12. Abbreviations: GSH: reduced gluthathione; GSSG: oxidised glutathione; Cys: cysteine, CySS: cystine; HCys: homocysteine, HCySS: homocystine, * = *p* < 0.05.

**Figure 2:**
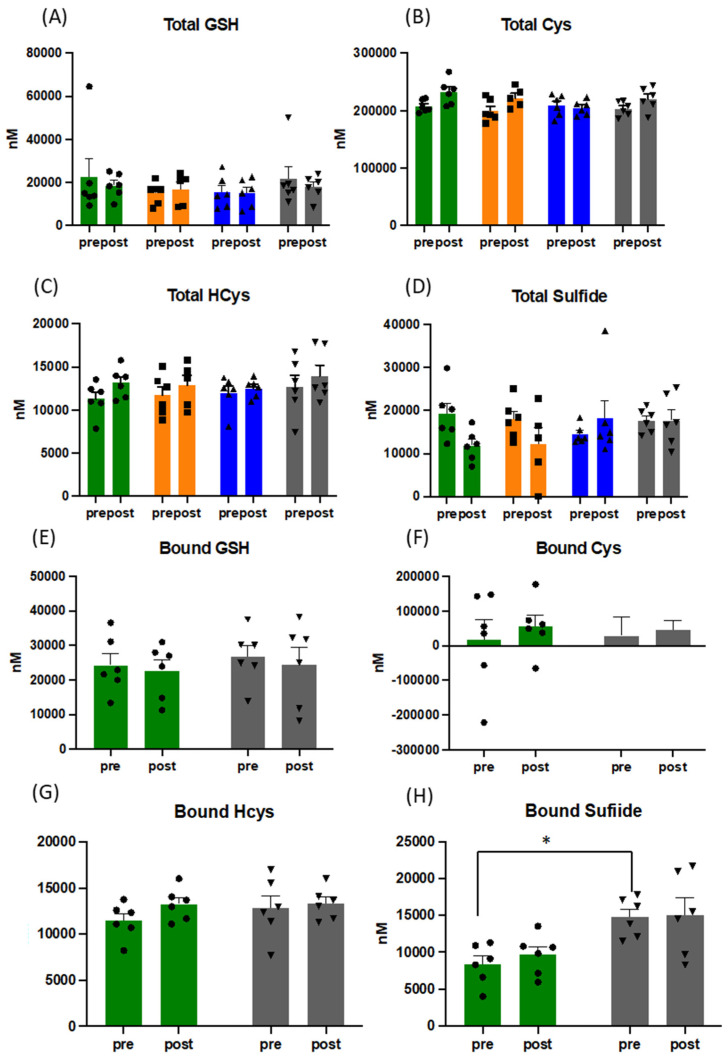
Pattern of response to changes in concentration of total and bound thiols in plasma (**A**–**H**). Data presented for before and after acute physiological challenge of a cardiopulmonary exercise test at each sampling point throughout the training intervention, presented as mean and SEM, with overlaid individual data (circles, squares and triangles; for the sake of direct comparison to free thiols, all concentrations are presented in [nM]). Green bars = baseline, orange bars = week 3, blue bars = week 6, and grey bars = week 12. Abbreviations: GSH: gluthathione; Cys: cysteine; HCys: homocysteine, * = *p* < 0.05.

At all sampling points, blood samples were taken before and after an acute exercise challenge in the form of a maximal, symptom-limited incremental CPET to assess changes to free and bound thiols, nitric oxide metabolism, lipid oxidation products, and reducing capacity of plasma. Whereas total free thiol (TFT) concentrations dropped marginally upon acute exercise circulating concentrations were not changed by the exercise intervention overall. Wide intra- and inter-subject variation in plasma thiol concentrations was observed in response to acute and repeated exercise (see Supplementary Figure S4), which seemed to reflect individual redox responses to the intervention. Figure 1 and Figure 2 depict the pattern of changes of individual constituents of the thiol metabolome over the course of the intervention period compared to that of baseline.

### 3.4. Acute and Long-Term Changes of the Thiol Metabolome in Response to Exercise

The acute changes of the redox response to exercise are complex and reflective of the oxidative challenge of exercise. Whilst there were no statistically significant changes in the thiol metabolomic responses, the heterogeneity of the levels pre- and post-exercise between different markers highlight the complexity of the integrative response (see Supplementary Figure S4). The plasma concentration of all forms of thiols (total = bound + free thiols) increased acutely with exercise for glutathione, cysteine, N-acetylcysteine, and marginally for homocysteine whereas it dropped for sulfide, indicative of net uptake and/or oxidation of the latter with exercise. Consistent with the literature [44], the cysteine and glutathione redox couples were not in equilibrium, with redox ratios changing into opposite direction. The directionality of reduced/oxidized homocysteine ratios changed from beginning to end of the exercise intervention whereas the quality of the response to exercise with other thiols showed little variation.

There were significant changes over the intervention period in the ratio of reduced (GSH) over oxidised glutathione (GSSG), when measured before CPET ((mean of 117 vs. 90), *p* = 0.029) (Figure 1), with maximal effects for the entire group peaking at week 6. Despite the expected increase in oxidative stress during acute exercise (confirmed by increased TBARS (mean of 4.6 vs. 5.95 pre and post CPET at baseline and mean of 6.1 vs. 6.95 pre and post CPET at week 12) and slightly lower total free thiol levels), GSH/GSSG ratios did not drop following acute exercise challenge, but rather increased due to a reduction in plasma GSSG concentrations. No such behaviour was observed for cysteine/cystine ratios (Figure 1). Oxidised homocysteine (HCyss) levels also significantly increased pre-CPET (*p* = 0.028), whereas total homocysteine levels increased only marginally with acute exercise. No obvious alterations in the patterns of reduced glutamylcysteine and cysteinylglycine were apparent over the course of the exercise intervention suggesting minor changes in glutathione synthesis and breakdown. Absolute amounts of bound thiols changed little for glutathione in response to acute exercise and tended to increase for cysteine and homocysteine, indicative of oxidative stress-induced (homo)cysteinylation of proteins. Whereas this pattern was maintained for the duration of the intervention, bound sulfide levels almost doubled, indicative of persulfide formation (sulfhydration) (see Figure 2), with persulfides demonstrated to contribute to protecting cells from ROS associated damage [45]. Persulfides are not picked up by the FRAP assay, indicating additional increases in overall reducing capacity.

### 3.5. Effects of Exercise on Nitric Oxide Metabolites, Lipid Oxidation Products and Reducing Capacity

**Figure 3:**
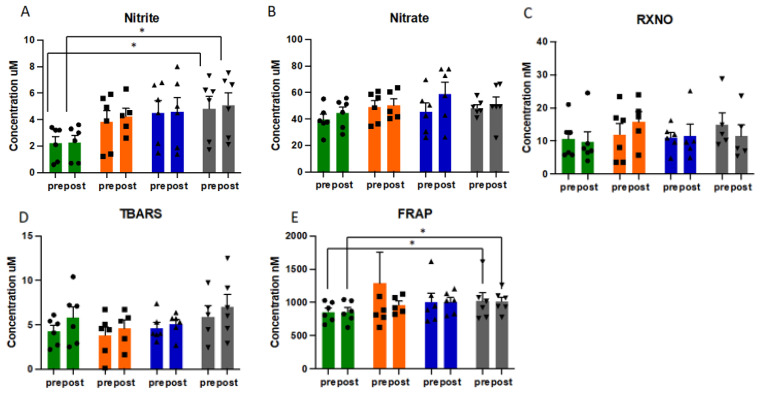
Pattern of redox responses to pre- and post-acute exercise challenge at each sampling point in study for plasma concentrations of nitrite (**A**), nitrate (**B**), nitroso species (**C**), TBARS (**D**) and FRAP (**E**), presented as mean and SEM, with individual data (circles, squares and triangles) overlaid. Data presented in µM or nM. Green bars = baseline, orange bars = week 3, blue bars = week 6, grey bars = week 12. Abbreviations: CPET: cardiopulmonary exercise test; RXNO: total nitroso species; TBARS: thiobarbituric acid reactive substances; FRAP: ferric reducing ability of plasma. * = *p* < 0.05.

Nitric oxide metabolism was significantly altered by exercise training, with distinct response patterns in each individual, demonstrating discrete adaptive responses in RNS readouts (Figure 3 and Figure S6). With steady-state concentrations of approx. 2 µM plasma nitrite concentrations were higher than what is typically observed in healthy individuals of comparable age, significantly (*p* = 0.028) increasing further over the course of the exercise intervention (Figure 3A). Nitrate and nitroso species (RXNO) levels did not significantly change. As expected, plasma lipid peroxidation, as assessed by TBARS, increased acutely with exercise and appeared to gradually rise over the 12-week intervention period (Figure 3D), but changes did not reach statistical significance. Despite the variability in response with individual markers of the thiol metabolome, overall antioxidant capacity of plasma, as assessed using FRAP, significantly increased following the exercise intervention, both pre- and post-acute exercise challenge (*p* = 0.028 for both pre- and post-CPET results) (Figure 3E).

### 3.6. Redox Status Is Associated with Improved Physical Fitness and Reduced Inflammation

**Figure 4:**
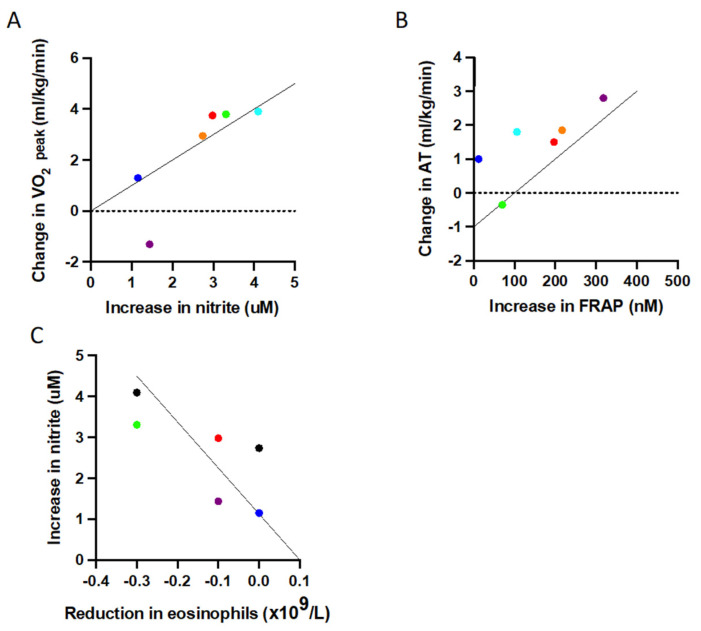
Significant correlations (Spearman’s rho correlation) between improvements in physical fitness, increased redox capacity and inflammation. (**A**) A greater increase in maximum oxygen uptake is associated with a greater increase in pre-CPET nitrite from baseline (r = 0.943, *p* = 0.019), (**B**) a greater increase in oxygen uptake at AT strongly correlates with a larger increase in FRAP (r = 0.886, *p* = 0.019), and (**C**) a greater increase in pre-CPET nitrite significantly correlates with a greater reduction in peripheral blood eosinophil levels (r = −0.837, *p* = 0.038). Abbreviations AT: (oxygen uptake at) anaerobic threshold; FRAP: ferric reducing ability of plasma.

The observed changes in fitness and reduction in inflammation following the exercise intervention correlated with increases in plasma nitrite concentration and overall antioxidant capacity (Figure 4). Specifically, there was a strong correlation between increases in fitness, as assessed by oxygen uptake at AT and increased antioxidant capacity following acute exercise challenge (r = 0.886, *p* = 0.019) (Figure 4A). There was also a strong correlation between the increase in nitrite (over the duration of the intervention, in samples taken before the acute exercise challenge) and increases in physical fitness, as assessed by oxygen uptake at peak exercise (r = 0.943, *p* = 0.005) (Figure 4B). The increase in nitrite (over the duration of the intervention, when sampled pre-CPET) also demonstrated a strong association with the reduction in eosinophil numbers following the exercise intervention (r = −0.837, *p* = 0.038) (Figure 4C). The changes in these redox measurements appeared to link the changes in fitness with the clinical improvements in inflammatory parameters, through to changes in symptom levels in these participants, as outlined in Supplementary Figure S5.

## 4. Discussion

It is increasingly appreciated that exercise interventions can improve symptom burden and quality of life in asthma [21], while also ameliorating systemic (and possibly airway) inflammation [20]. The mechanism through which these changes are mediated remained elusive. We here, not only offer further corroborative support for the beneficial effects of physical activity, but, for the first time, provide experimental evidence for a link between exercise-induced plasma nitrite elevations, changes in extracellular redox status and reduction in inflammatory burden in asthma. The stability of body mass index provides some reassurance that these changes are not merely a result of reduced adiposity-driven inflammation. Demonstration of the disease-modifying benefits of exercise in asthma should go a long way in increasing uptake in patients with greater perceived barriers to activity [46]. There is support for our findings from work in other chronic inflammatory diseases, summarised in a review that suggests exercise interventions exert antioxidant effects [47]. However, to the best of our knowledge, our study is the first to use in-depth, multilevel redox profiling (including group-specific biochemical readouts of relevance to clinical outcome) in a longitudinal manner, and as such, offers a number of novel insights.

There are suggestions from small studies that exercise exerts a systemic anti-inflammatory effect in asthma, as summarised in a recent review [21]. Our study provides support for this, with a specific impact on asthma relevant IL-5/eosinophil pathways. Of note, the response to an equivalent degree of redox stress, in the form of a personalised and metabolically controlled exercise intervention demonstrates wide interindividual variation in patients with asthma. A similar heterogeneity in response to a comparable metabolic challenge was previously reported in healthy individuals [18]. Thus, interindividual differences in response to exercise training may not be disease specific. Despite these variabilities, following the exercise intervention and prior to an acute exercise challenge, there was an overall decrease in the ratio of reduced over oxidised glutathione combined with an increase in oxidised homocysteine. The change in the GSH/GSSG ratio towards a more oxidised status is consistent with the significant reduction in GSH/GSSG ratio that was demonstrated in bronchial samples in relation to asthma severity and control (with these studies demonstrating an increase in disease severity with increased levels of oxidised glutathione) [48,49]. These studies looked at GSH and GSSG within bronchoalveolar lavage fluid, whereas the changes in our study were documented in blood plasma. Contrary to our expectations, GSH/GSSG ratios increased rather than further decreased, after exercise later in the training programme. These directionally opposing changes suggest a greater capacity for reduction (instead of greater oxidative stress) as a direct result of the exercise training programme. These seemingly counterintuitive changes may be explained by the need for redox balance at the whole-body level. There is supportive evidence for this explanation in the literature, with demonstration of opposing changes in glutathione redox status within plasma and erythrocytes in critically ill patients [50] and in healthy individuals subjected to acute aerobic exercise [51]. Alternatively, the lower GSSG levels after exercise may be reflective of an increased capacity for reduction in skeletal muscle. Our data, demonstrating a reduction in GSH to GSSG ratio in the apparent absence of an increased utilisation of GSH (see Figure 1) may therefore reflect greater reducing capacity following exercise training. Similarly, the increase in oxidised homocysteine (HCyss) observed in the present study was not in a direction expected with the hypothesised of increased resilience to oxidised stress. Exercise-induced changes in plasma total homocysteine levels were reported previously, however, the magnitude and direction of those changes are inconsistent and show sexual dimorphism [52,53,54]. While patients with chronic respiratory diseases typically have higher homocysteine levels than age-matched controls and elevated levels are a recognised risk factor for cardiovascular disease [55,56], it is unlikely that the beneficial effects of exercise in asthma are associated with increased cardiovascular risk. Rather, they may reflect the changes in extracellular redox status documented since HCys is at a critical branching point between the methionine recycling and trans-sulfuration pathway, and the latter is under redox control [57,58].

The lack of association between FeNO and nitrite levels are unsurprising as no clear link between the two was previously shown [59,60]. In contrast to FeNO and nitrite levels in exhaled breath condensate (EBC), levels of EBC nitrate were reported to relate to asthma control [61] and with one exception, EBC nitrate levels tended to increase in the present study (Figure S3). Nitrosative stress levels in the airways, as assessed through measurement of nitroso products in EBC, did not demonstrate consistent changes. This may be reflective of the moderate number of participants, and whilst there was no statistically significant change, those levels appear to gradually decrease upon exercise training (with transient rises in two out of four individuals). This would be consistent with increased nitrosative chemistry in inflammation and the interpretation that pulmonary inflammatory status decreases with exercise training. This conjecture needs validation and further investigation in larger mechanistic studies.

The increase in overall antioxidant capacity of the extracellular fluid compartment, as measured by the ferric reducing ability of plasma (FRAP), following the exercise intervention is also noteworthy. There is demonstration that FRAP is reduced in acute asthma exacerbations [11]. Many of the irritants that drive exacerbation of asthma, such as microbial and allergenic stimuli, act to increase ROS. Greater ability to buffer ROS through an increase in antioxidant capacity would theoretically increase an individual’s tolerance to these stressors. Orthogonal to the enhanced reducing ability of plasma is the increase in bound sulfide. Persulfides would not be expected to be picked up by the FRAP assay but are known to have potent antioxidant activity. This aspect of our thiol metabolome analysis would seem to merit further investigation with identification of the particular species formed during exercise interventions in future studies.

In the vasculature, nitrite is generated by shear-stress-induced stimulation of endothelial NO production, predicts exercise capacity, and its formation is compromised in patients with cardiovascular risk factors [31,62]. The nitrite changes observed in the present study are worthy of more detailed discussion since they seem to mechanistically link exercise to the beneficial effects on inflammation and quality of life—they also shift the centre of attention from oxidative stress to a biochemical messenger endowed with redox-regulatory capacity. Firstly, levels of nitrite throughout the intervention were higher than we have seen in healthy individuals in response to an acute exercise challenge [18]. This is consistent with other work, demonstrating higher levels in patients with both stable and exacerbating asthma compared to healthy controls [63,64]. However, the conclusion the authors of this work reached was that the higher nitrite concentrations reflected an increase in oxidative stress due to inflammation. Our data suggest the relationship between NO metabolism and inflammation is more complex and associated with changes in the overall redox landscape. The relationship is further complicated by the impact exercise has on the NO pathway, with exercise shown to increase nitrite in both young and older adults [65,66]. An alternative explanation to understand these complexities may be found in the multifactorial role nitrite plays in redox chemistry in the lung [9]. Asthmatic airways present a more acidic environment, which encourages nitrite protonation to NO, with less efficient formation of nitrite via NO oxidation [9]. A reduction in systemic inflammation following the exercise intervention may result in reduced airway acidity, with a shift in balance towards nitrite formation. Additionally, nitrite is consumed by leukocyte peroxidases, including eosinophil peroxidase secreted from activated eosinophils [67]. If exercise intervention reduces the numbers of eosinophils, then secretion of eosinophil peroxidase may also be reduced, with subsequent increases in nitrite levels. Moreover, increased nitrite levels may promote formation of the endogenous bronchodilator S-nitrosoglutathione (GSNO) [9,68], facilitating bronchodilation. However, it is also conceivable that the nitrite increases we observed in our asthma cohort do not at all originate from shear-stress-induced eNOS stimulation followed by oxidation of NO in the microcirculation, but rather originate from an exercise-induced biotransformation of nitrate in skeletal muscle [69,70]. This tissue compartment has recently been identified as an important storage pool for endogenously produced and dietarily ingested nitrate, and exercise-induced metabolic hypoxia may promote conversion of nitrate to nitrite. Indeed, nitrate reduction to nitrite (and further to NO) was demonstrated in hypoxic/anoxic tissue [71,72]. Such a mechanism would also be consistent with the observation that high endogenous nitrite levels are associated with superior exercise capacity, independent of endothelial function, in highly trained athletes [73].

It is conceivable that the exercise-induced increases in plasma nitrite levels we observed in the present study are indeed causally linked to the improved plasma antioxidant capacity. If true, the question arises by what mechanism nitrite exerts these effects. This is also of interest considering that a recent placebo-controlled proof-of-principle trial with nebulised sodium nitrite (15 mg given twice daily for 12 weeks) in asthma patients demonstrated modest improvements in FEV1 and reduced exacerbations, albeit without affecting asthma symptoms [74]. Exogenous nitrite is often believed to act as a reservoir for NO [75]. However, nitrite is not only an oxidative breakdown product of NO that can be recycled under appropriate conditions but a signalling molecule in its own right [76]. Besides acting as an oxidant, promoting blood pressure lowering via PKG-dimerization [77], nitrite modulates the expression of heme oxygenase-1 (HO-1) in a variety of tissues [76]. HO-1 has antioxidant effects by generating biliverdin and carbon monoxide (CO), modulating mitochondrial and metabolic activity [78] and is also an important regulator of aerobic activity in skeletal muscle [79]. Altogether, this would seem to provide a coherent picture by which exercise promotes the release of nitrite from stored nitrate in muscle, triggering (possibly Nrf2-mediated) oxidant-driven systemic antioxidants effects to downregulate chronic inflammation in asthma and possibly other disease processes accompanying inflammation.

### Study Limitations

Our study has strengths and weaknesses, but the data must be interpreted as exploratory due to the small cohort of patients longitudinally profiled. Importantly, our main clinical endpoint was improvement in asthma control, which we found to be statistically significant. We, therefore, undertook other exploratory analyses to gain first insights into potential mechanisms associated with this effect. However, small numbers increase the risk of both type-1 errors due to repeated statistical testing within the dataset, and of type-2 errors. The high dropout rate and per-protocol analysis may bias results towards responders. In defence of this, participants who did not complete the exercise intervention also demonstrated improvements in symptoms and quality of life up to the point of dropout (data not shown), and reasons for dropout were not reported as study-related (see Figure S1). Another limitation is that the cohort was comprised only of female patients. There is a female predominance in difficult asthma which may partly justify this cohort composition [43]. Furthermore, obese females comprise a large proportion of patients with difficult asthma [43], with obesity adding additional metabolic and oxidative burden [80]. Females with adult-onset asthma also tend to be nonatopic, reducing their treatment options, and are therefore a group which would benefit from new treatment strategies [43]. Additionally, demonstration of redox regulation-driven responses to exercise in a male-only group suggest our results could be extrapolated to a mixed sex cohort [81]. The lack of a control group presents difficulty in ascertaining that the results obtained herein reflect the natural variability over time rather than changes as a result of the exercise intervention; specifically, it is difficult to exclude an increased adherence with prescribed medication as a confounder. However, if participants had increased adherence with inhaled corticosteroids, then an increase, as opposed to the demonstrated reduction in white cell count, would be expected. The lack of a healthy control group raises the question as to whether these changes are specific to asthma or whether they are a more generalised response to the exercise intervention. Whilst we cannot exclude that dietary variation may have contributed to some of the changes observed, all samples were taken at similar times of the day, with participants avoiding nitrate-rich foods in preparation for FeNO sampling. Finally, the responses demonstrated herein are systemic, and future work may need to investigate the contribution of blood cells to the antioxidant effects and lung inflammatory responses more specifically. Further work should also include investigation into the relationship between eosinophil peroxidase and increasing nitrite levels. Despite these limitations, this exploratory work demonstrates novel insight into the complex responses of the redox metabolome to exercise intervention in chronic inflammatory disease. The significant changes demonstrated offer insight into potential mechanisms of exercise intervention in asthma that require investigation in greater depth in dedicated future studies.

## 5. Conclusions

Exercise is increasingly appreciated to confer improvements in quality of life and symptoms in patients with chronic disease, alongside improvements in inflammation. The mechanisms driving these responses remain unclear [82]. In parallel, there is growing evidence to support the benefit of exercise in strengthening the resilience of the redox regulation system at the whole-body level. This exploratory work highlights the relevance of exercise-mediated increases in redox resilience as a potential mechanism to understand the clinical benefits of exercise in asthma. This hypothesis requires further investigation to confirm the findings demonstrated here, in a larger group of patients with both healthy and non-exercised controls. Improved understanding of exercise-mediated modulation of complex redox pathways offers the opportunity for greater personalisation of treatment and potential for identification of new treatment targets.

## Figures and Tables

**Table 1:** Participant demographics and medications.

Characteristic	Number (%) or Median [IQR]
Female sex	6 (100)
Age (years)	31.3 ± 10
Never smoker	5 (83)
BMI (kg/m^2^)	27.6 [22.26, 30.66]
Peripheral blood eosinophil count	0.25 [0.2, 0.73]
FeNO (ppb)	50.75 [27.25, 93]
Co-morbidities	Number (%)
Atopy	5 (83)
Anxiety and depression	2 (33)
Urticaria and angioedema	1 (16%)
Anaphylaxis	1 (16%)
Dysfunctional breathing	1 (16%)
Pulmonary Function	Median [IQR]
FEV1 % predicted	89 [78.75, 94.5]
FVC % predicted	100.5 [91.75, 1.3]
FEV1/FVC	77 [73.75, 82.25]
FEF 25–75 % predicted	68.5 [31.30, 85.25]
% change FEV1 post BD	5 [3, 13.25]
Asthma Medication	Number (%) or mean ± SD
on ICS	4 (66%)
ICS dose (BDP equivalent µg/day)	483 ± 371
on LABA	2
LABA dose (formoterol equivalent µg/day)	7.2 ± 10.73
on Montelukast	1 (16%)

Presented as number and percentage, or median and IQR. Abbreviations: BD: bronchodilator; BDP: beclomethasone dipropionate; BMI: body mass index; FEF 25–75: mid-expiratory flow rate FeNO: fractional exhaled nitric oxide; FEV1: forced expiratory volume in 1 s; FVC: forced vital capacity; ICS: inhaled corticosteroid; LABA: long-acting bronchodilator.

**Table 2:** Body composition and aerobic capacity.

Variables	Baseline Median (IQR)	Post Intervention Median (IQR)	*p* Value
Anthropometric Values			
BMI Height (cm) Weight (kg)	27.7 (22.3, 30.7) 171.5 (164.3, 172.1) 78.2 (58.8, 91.8)	27.6 (22, 29.6) n.d. n.d.	0.136 - -
Fat-free mass (%)	62.9 (58.3, 67.5)	60.7 (56.2, 67.5)	1
Fat mass (%)	36.6 (32.5, 41.7)	36.8 (32.5, 42.1)	0.528
Visceral Adipose Tissue (litres)	0.83 (0.45, 1.23)	0.74 (0.16, 1)	0.465
Skeletal Muscle Mass (kg)	23 (18.8, 27.2)	23 (18.3, 27.2)	0.463
Aerobic Capacity			
VO2 peak (mL/kg/min)	21.6 (19, 28)	25 (20, 31.5)	0.058 *
Anaerobic Threshold (mL/kg/min) Maximum Workload (watts)	10.4 (9.4, 14) 161 (135, 205)	12.6 (10, 15.6) 186 (172, 235)	0.046 * 0.028 *

* = statistically significant as assessed by Wilcoxon signed rank test. Abbreviations: BMI: body mass index; IQR: interquartile range; n.d: not determined; VO2 peak: maximal oxygen uptake.

**Table 3:** Symptom scores and quality of life scores between baseline and week 12.

Variable	Baseline Median (IQR)	Post 12-Week Intervention Median (IQR)	*p* Value
ACQ 6 Score	2 (1.8, 2.6)	1.2 (0.8, 1.5)	0.028 *
AQLQ Total Score	4.8 (4.1, 5.3)	5.8 (5.5, 6.3)	0.046 *
AQLQ Symptoms Domain	4.5 (3.8, 5)	5.8, 5.1, 6.3)	0.046 *
AQLQ Activity Domain	5.3 (4.4, 6)	6.1 5.7, 6.8)	0.116
AQLQ Emotions Domain	4.8 (2.7, 5.3)	6.1 (5.3, 6.5)	0.027 *
AQLQ Environmental Domain	4.5 (3.6, 5.4)	5.6 (4.9, 6.6)	0.043 *

Statistical assessment by Wilcoxon signed rank test, for (ACQ 6 Score) asthma control questionnaire, (AQLQ Total Score) asthma quality of life questionnaire, (AQLQ Symptoms Domain) asthma quality of life questionnaire symptom domain, (AQLQ Activity Domain) asthma quality of life activity domain, (AQLQ Emotions Domain) asthma quality of life emotional domain, and (AQLQ Environmental Domain) asthma quality of life questionnaire environmental domain. * = statistically significant median and IQR for each patient. IQR: interquartile range.

**Table 4:** Assessment of inflammation.

Variables	Baseline Median (IQR)	Post Intervention Median (IQR)	*p* Value
Airway Inflammation			
FeNO (ppb)	50 (27, 93)	35 (19, 94)	0.753
Clinical inflammatory markers			
WCC × 10^9^/L	7.2 (5.6, 9.1)	6.7 (4.0, 7.5)	0.046 *
Neutrophils × 10^9^/L	6.7 (2.7, 5.8)	3.9 (1.9, 4.6)	0.054
Eosinophils × 10^9^/L	0.25 (0.2, 0.73)	0.2 (0.18, 0.43)	0.007 *
Lymphocytes × 10^9^/L	2.2 (1.6, 2.7)	1.8 (1.7, 2.2)	0.049 *
Monocytes × 10^9^/L	0.5 (0.38, 0.55)	0.45 (0.45, 0.53)	0.414
CRP mg/L	3 (1.5, 6.3)	1 (0.75, 6.8)	0.340
Cytokines (pg/mL)			
CCL11/eotaxin	173.0 (138.5, 214.0)	153.97 (134.0, 180.1)	0.046 *
IL-5	7.24 (6.39, 10.33)	5.34 (4.04, 7.37)	0.046 *
TNFα	15.63 (9.75, 15.63)	9.96 (5.19, 12.53)	0.046 *
IFNγ	17.89 (10.87, 20.32)	11.9 (7.48, 12.91)	0.046 *
IL1-ra	644.3 (328.0, 1077.8)	469.9 (307.1, 900.8)	0.116
IL-6	8.32 (6.35, 12.48)	6.60 (5.40, 8.74)	0.115
IL-10	79.20 (65.89, 96.72)	72.92 (58.80, 78.7)	0.173
IL-13	66. 81 (58.95, 76.23)	55.72 (48.70, 64.85)	0.141

Statistical assessment by Wilcoxon or Friedman test, * = statistically significant. Abbreviations: IQR: interquartile range; CRP: C reactive protein; IFN: interferon; IL: interleukin; pg/mL; picograms/millilitre; TNF: tumour necrosis factor; WCC: white cell count.

## Data Availability

Data sharing not applicable.

## Supplementary Materials

The supplementary materials are available online at https://www.mdpi.com/article/10.3390/antiox10121926/s1.
