# Supplementary material for: Exercise Training Induces a Shift in Extracellular Redox Status with Alterations in the Pulmonary and Systemic Redox Landscape in Asthma
Source: Antioxidants (Basel). 2021 Nov 30;10(12):1926. doi: 10.3390/antiox10121926 (PMC8750917; doi:10.3390/antiox10121926)
Supplement: Supplementary file 1 [file antioxidants-10-01926-s001.zip › antioxidants-1458341-supplementary.pdf]

Table S1. Pre-bronchodilator spirometry between baseline and week 12, as assessed by Wilcoxon signed rank test.

**Variables**	**Baseline** median (IQR)	**Post intervention** median (IQR)	**p value**
Lung function			
Pre BD FEV1 (litres)	2.91 (2.39, 3.37)	3.2 (2.5, 3.6)	0.173
Pre BD FVC (litres)	3.98 (3.12, 4.32)	4.29 (3.36, 4.42)	**0.028***
FEV1/FVC ratio	77 (74, 82)	77 (72, 82)	0.916
% change FEV1 post BD	8 (3.8, 13.3)	3 (2.3, 6.3)	**0.043***

**= statistically significant.*

Abbreviations: BD; bronchodilator, FEV1; forced expiratory volume in 1 second, FVC; forced vital capacity, IQR; interquartile range

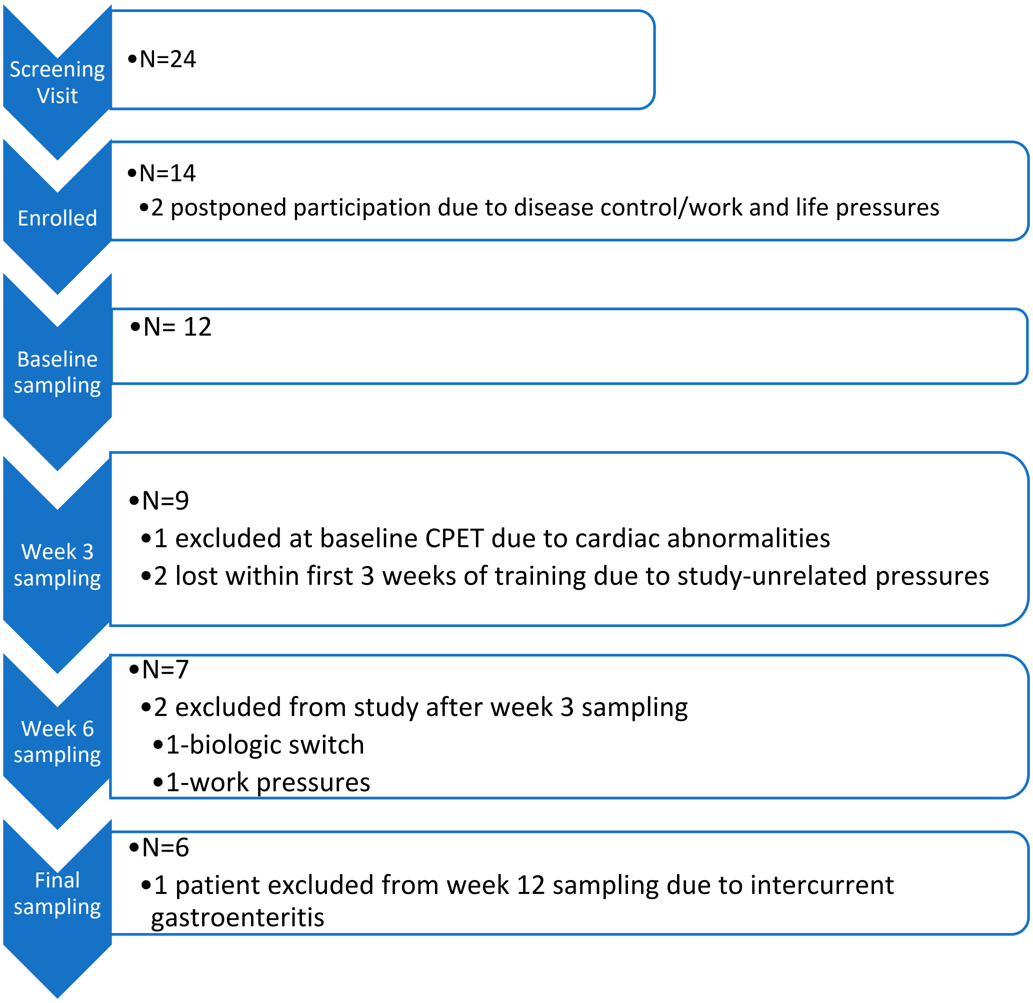

Figure S1. Flow chart of patient enrolment and sampling, including numbers and reasons for withdrawal/exclusion

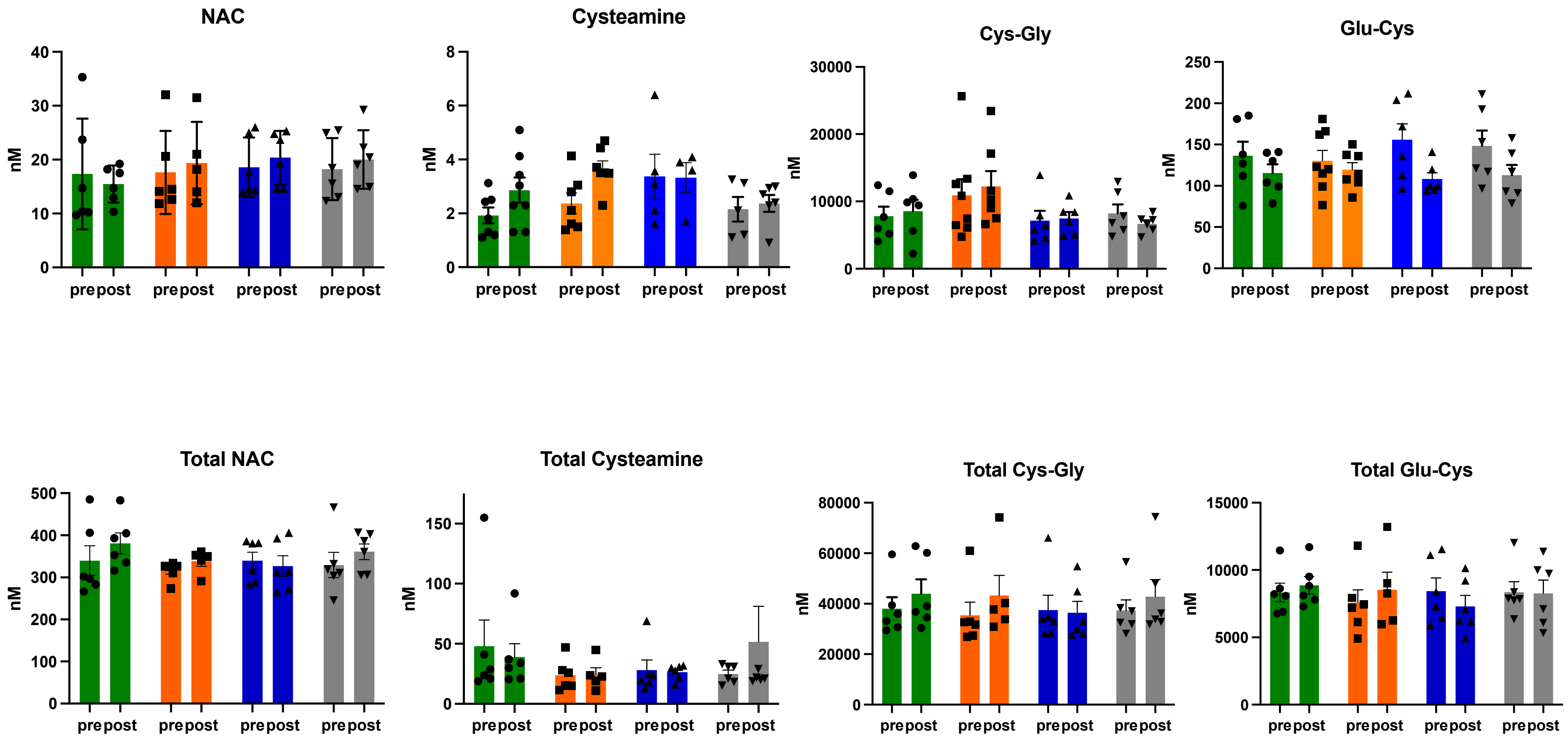

Figure S2 Changes in the pattern of responses of the free and total redox metabolome. Data presented for n=6 before and after acute physiological challenge of a cardiopulmonary exercise test, presented as mean and SEM, with individual data plotted. Data presented in nM. Green bars= baseline, orange bars= week 3, blue bars = week 6 and grey bars =week 12. Abbreviations: NAC; N-acetylcysteine, Cys-Gly; cysteinylglycine; Glu-Cys; L-Glutamyl-L-Cysteine

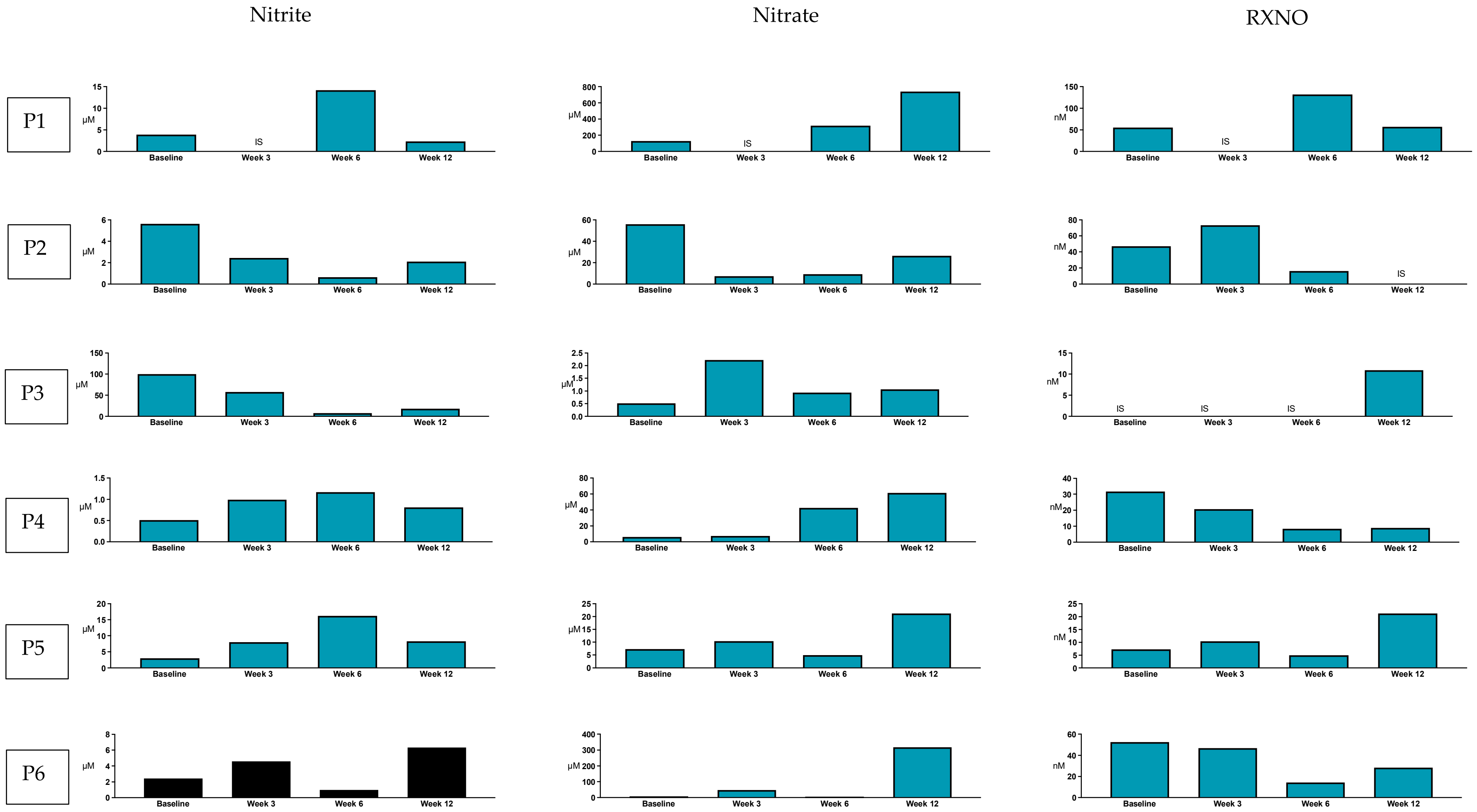

Figure S3. Pattern of airways nitroso-metabolic responses to exercise training, prior to acute exercise challenge, at baseline, week, 3, 6 and post intervention. Data presented for each patient (P1-6), units nitrite and nitrate = μM, RXNO=nM. Blue bar charts = nitrite, turquoise = nitrate and magenta = RXNO .Abbreviations RXNO; other nitrosospecies. IS = insufficient sample to quantify

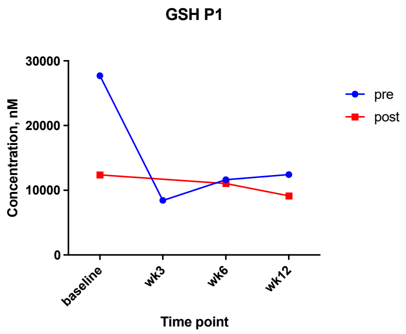
GSH P1
30000
20000
10000
0
Concentration, nM
baseline
wk3
wk6
wk12
Time point
pre
post

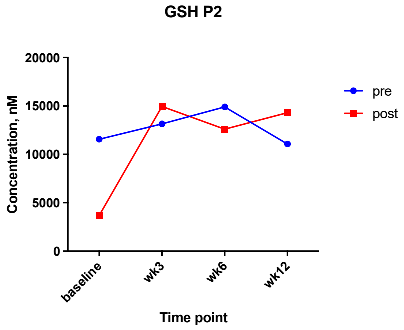
GSH P2
20000
15000
10000
5000
0
Concentration, nM
baseline
wk3
wk6
wk12
Time point
pre
post

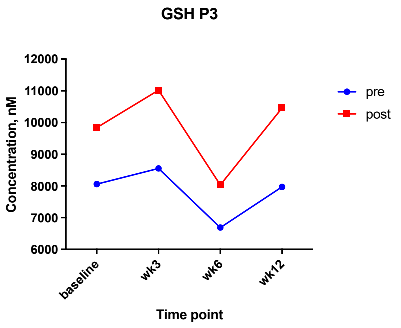
GSH P3
12000
11000
10000
9000
8000
7000
6000
Concentration, nM
baseline
wk3
wk6
wk12
Time point
pre
post

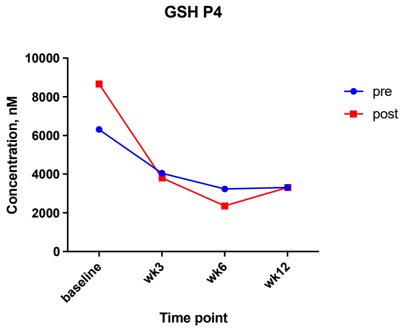
GSH P4
10000
8000
6000
4000
2000
0
Concentration, nM
baseline
wk3
wk6
wk12
Time point
pre
post

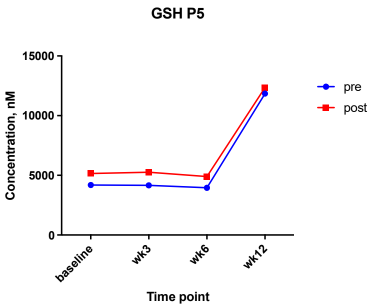
GSH P5
15000
10000
5000
0
Concentration, nM
baseline
wk3
wk6
wk12
Time point
pre
post

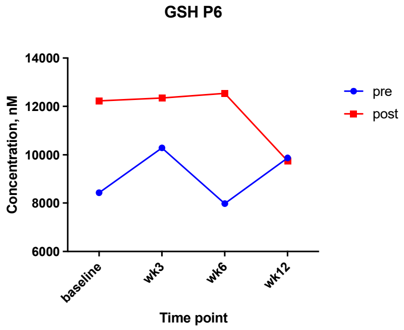
GSH P6
14000
12000
10000
8000
6000
Concentration, nM
baseline
wk3
wk6
wk12
Time point
pre
post

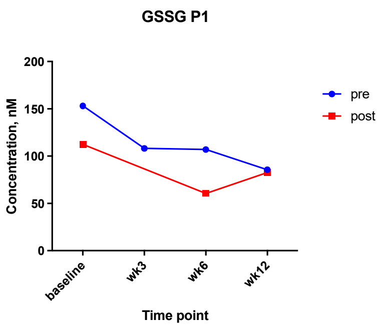
GSSG P1
Concentration, nM
0
50
100
150
200
baseline
wk3
wk6
wk12
Time point
pre
post

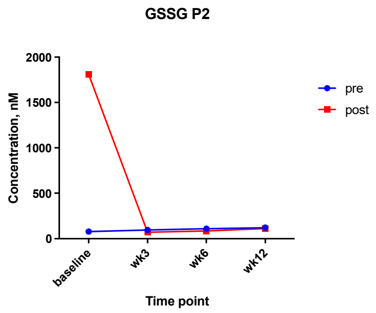
GSSG P2
Concentration, nM
0
500
1000
1500
2000
baseline
wk3
wk6
wk12
Time point
pre
post

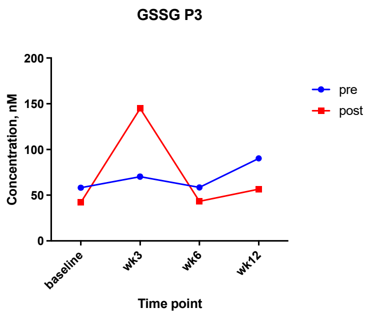
GSSG P3
Concentration, nM
0
50
100
150
200
baseline
wk3
wk6
wk12
Time point
pre
post

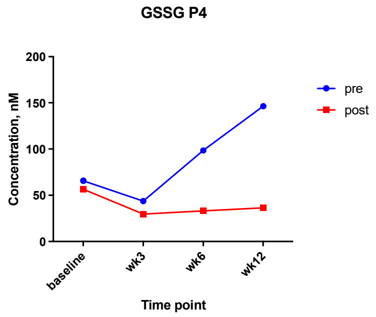
GSSG P4
Concentration, nM
0
50
100
150
200
baseline
wk3
wk6
wk12
Time point
pre
post

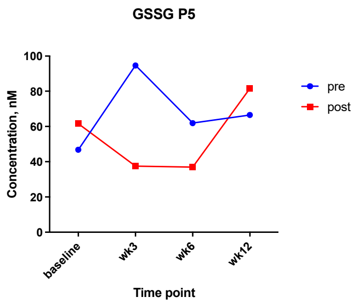
GSSG P5
Concentration, nM
0
20
40
60
80
100
baseline
wk3
wk6
wk12
Time point
pre
post

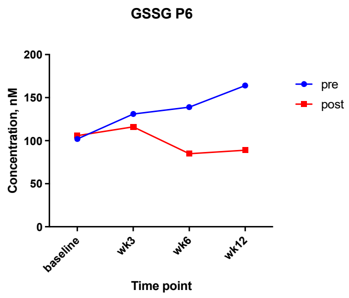
GSSG P6
Concentration, nM
0
50
100
150
200
baseline
wk3
wk6
wk12
Time point
pre
post

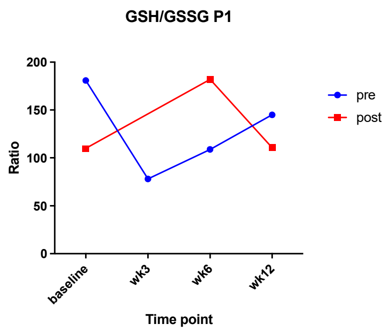
GSH/GSSG P1
Ratio
200
150
100
50
0
baseline
wk3
wk6
wk12
Time point
pre
post

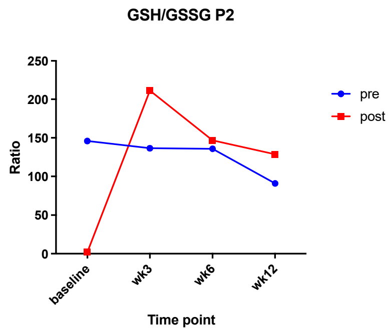
GSH/GSSG P2
Ratio
250
200
150
100
50
0
baseline
wk3
wk6
wk12
Time point
pre
post

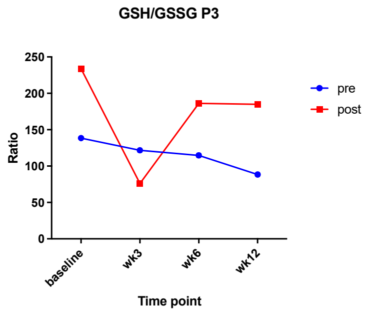
GSH/GSSG P3
Ratio
250
200
150
100
50
0
baseline
wk3
wk6
wk12
Time point
pre
post

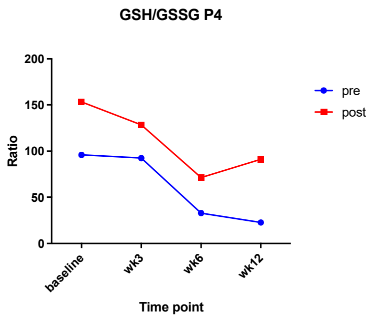
GSH/GSSG P4
Ratio
200
150
100
50
0
baseline
wk3
wk6
wk12
Time point
pre
post

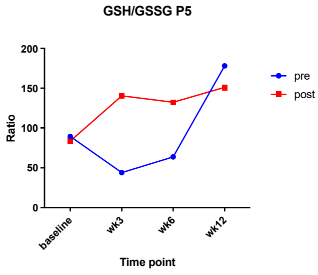
GSH/GSSG P5
Ratio
200
150
100
50
0
baseline
wk3
wk6
wk12
Time point
pre
post

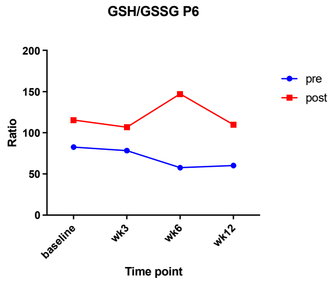
GSH/GSSG P6
Ratio
200
150
100
50
0
baseline
wk3
wk6
wk12
Time point
pre
post

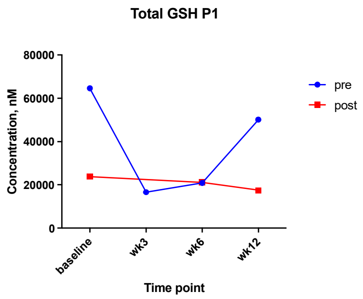
Total GSH P1
Concentration, nM
80000
60000
40000
20000
0
baseline
wk3
wk6
wk12
Time point
pre
post

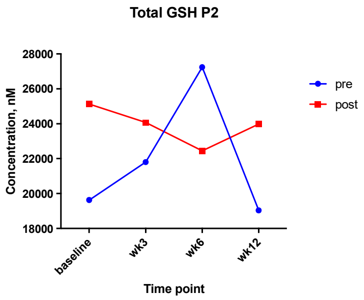
Total GSH P2
Concentration, nM
28000
26000
24000
22000
20000
18000
baseline
wk3
wk6
wk12
Time point
pre
post

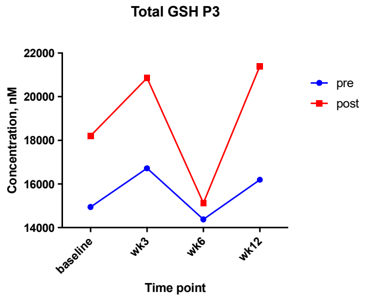
Total GSH P3
Concentration, nM
22000
20000
18000
16000
14000
baseline
wk3
wk6
wk12
Time point
pre
post

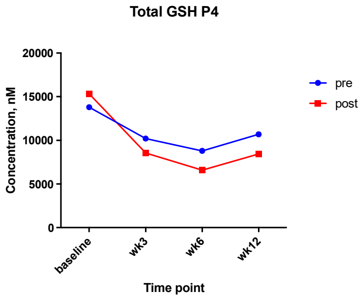
Total GSH P4
Concentration, nM
20000
15000
10000
5000
0
baseline
wk3
wk6
wk12
Time point
pre
post

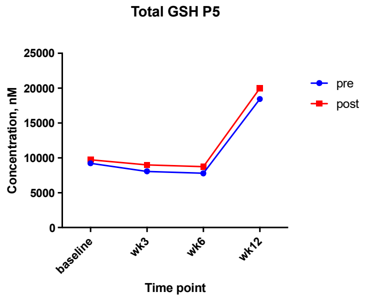
Total GSH P5
Concentration, nM
25000
20000
15000
10000
5000
0
baseline
wk3
wk6
wk12
Time point
pre
post

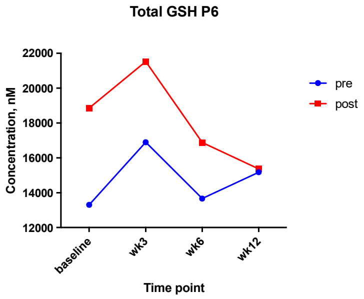
Total GSH P6
Concentration, nM
22000
20000
18000
16000
14000
12000
baseline
wk3
wk6
wk12
Time point
pre
post

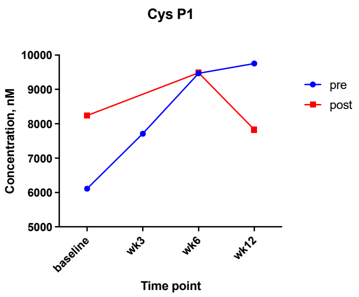
Cys P1
Concentration, nM
10000
9000
8000
7000
6000
5000
baseline
wk3
wk6
wk12
Time point
pre
post

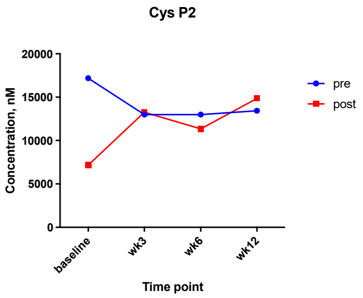
Cys P2
Concentration, nM
20000
15000
10000
5000
0
baseline
wk3
wk6
wk12
Time point
pre
post

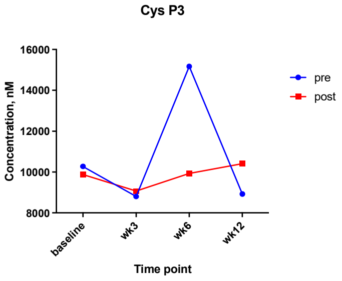
Cys P3
Concentration, nM
16000
14000
12000
10000
8000
baseline
wk3
wk6
wk12
Time point
pre
post

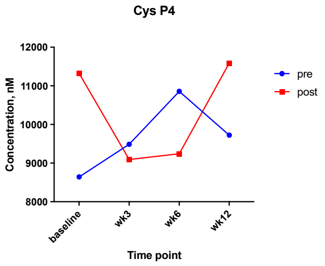
Cys P4
Concentration, nM
12000
11000
10000
9000
8000
baseline
wk3
wk6
wk12
Time point
pre
post

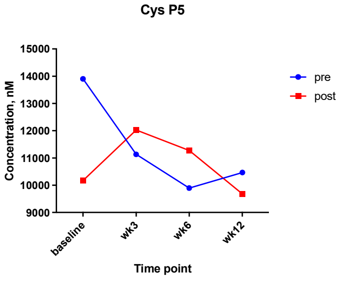
Cys P5
Concentration, nM
15000
14000
13000
12000
11000
10000
9000
baseline
wk3
wk6
wk12
Time point
pre
post

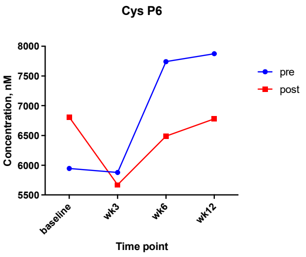
Cys P6
Concentration, nM
8000
7500
7000
6500
6000
5500
baseline
wk3
wk6
wk12
Time point
pre
post

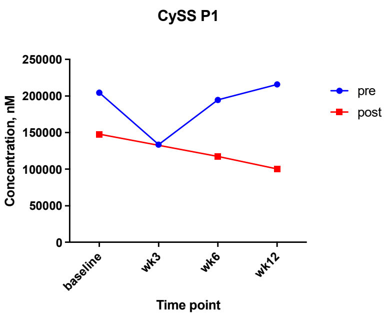
CySS P1
250000
200000
150000
100000
50000
0
Concentration, nM
baseline
wk3
wk6
wk12
Time point
pre
post

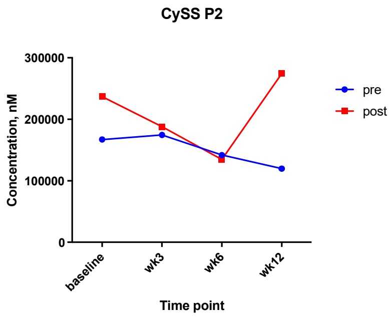
CySS P2
300000
200000
100000
0
Concentration, nM
baseline
wk3
wk6
wk12
Time point
pre
post

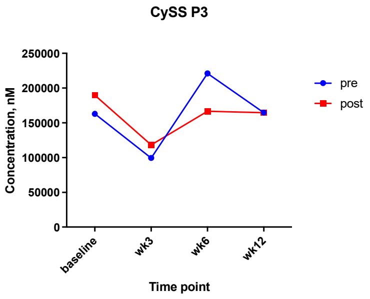
CySS P3
250000
200000
150000
100000
50000
0
Concentration, nM
baseline
wk3
wk6
wk12
Time point
pre
post

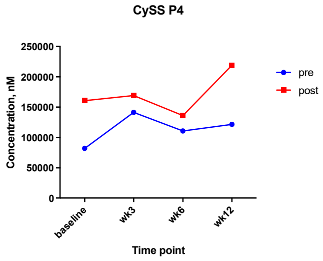
CySS P4
250000
200000
150000
100000
50000
0
Concentration, nM
baseline
wk3
wk6
wk12
Time point
pre
post

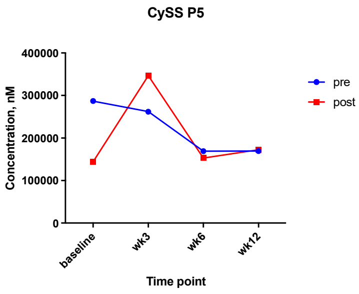
CySS P5
400000
300000
200000
100000
0
Concentration, nM
baseline
wk3
wk6
wk12
Time point
pre
post

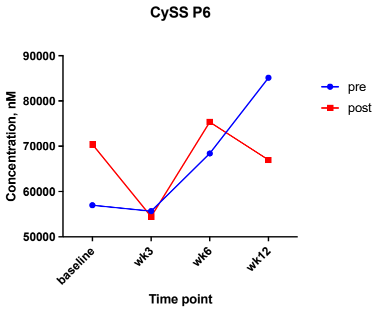
CySS P6
90000
80000
70000
60000
50000
Concentration, nM
baseline
wk3
wk6
wk12
Time point
pre
post

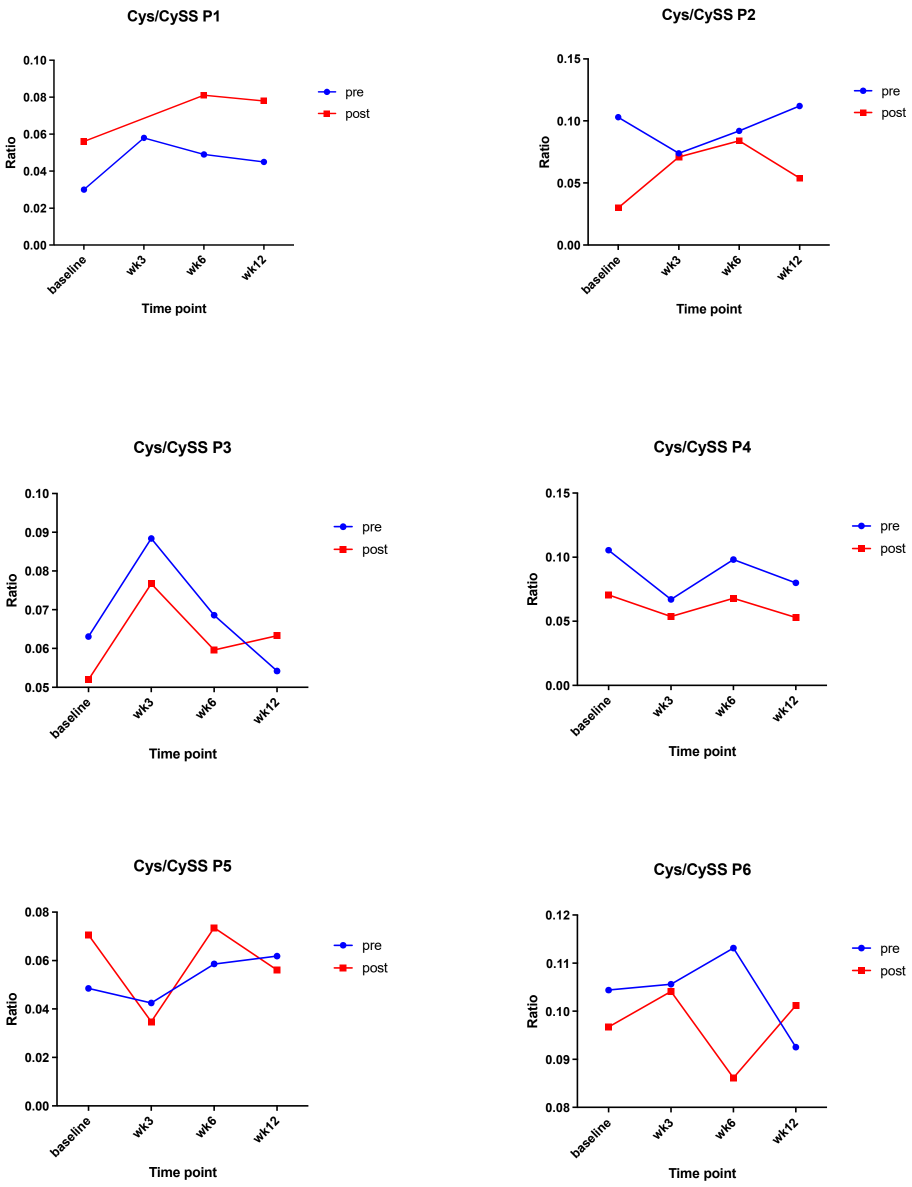
Cys/CySS P1
Ratio
0.00
0.02
0.04
0.06
0.08
0.10
baseline
wk3
wk6
wk12
Time point
pre
post
Cys/CySS P2
Ratio
0.00
0.05
0.10
0.15
baseline
wk3
wk6
wk12
Time point
pre
post
Cys/CySS P3
Ratio
0.05
0.06
0.07
0.08
0.09
0.10
baseline
wk3
wk6
wk12
Time point
pre
post
Cys/CySS P4
Ratio
0.00
0.05
0.10
0.15
baseline
wk3
wk6
wk12
Time point
pre
post
Cys/CySS P5
Ratio
0.00
0.02
0.04
0.06
0.08
baseline
wk3
wk6
wk12
Time point
pre
post
Cys/CySS P6
Ratio
0.08
0.09
0.10
0.11
0.12
baseline
wk3
wk6
wk12
Time point
pre
post

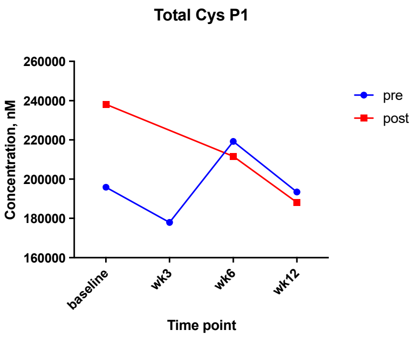
Total Cys P1
Concentration, nM
260000
240000
220000
200000
180000
160000
baseline
wk3
wk6
wk12
Time point
pre
post

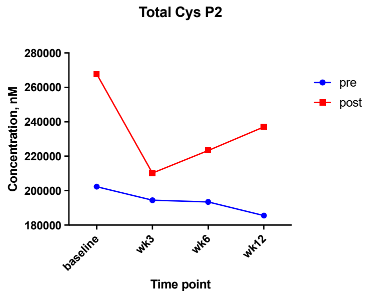
Total Cys P2
Concentration, nM
280000
260000
240000
220000
200000
180000
baseline
wk3
wk6
wk12
Time point
pre
post

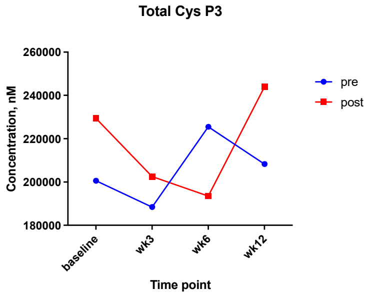
Total Cys P3
Concentration, nM
260000
240000
220000
200000
180000
baseline
wk3
wk6
wk12
Time point
pre
post

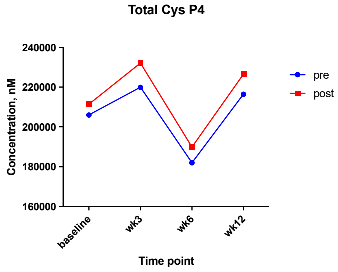
Total Cys P4
Concentration, nM
240000
220000
200000
180000
160000
baseline
wk3
wk6
wk12
Time point
pre
post

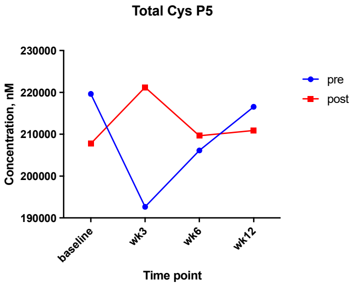
Total Cys P5
Concentration, nM
230000
220000
210000
200000
190000
baseline
wk3
wk6
wk12
Time point
pre
post

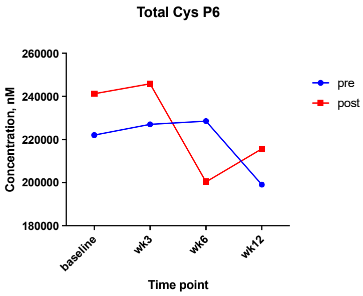
Total Cys P6
Concentration, nM
260000
240000
220000
200000
180000
baseline
wk3
wk6
wk12
Time point
pre
post

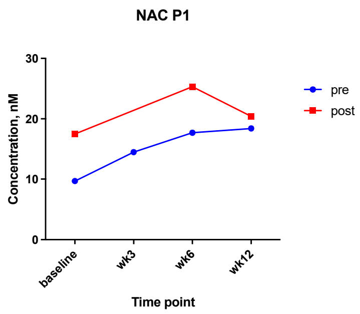
NAC P1
Concentration, nM
30
20
10
0
baseline
wk3
wk6
wk12
Time point
pre
post

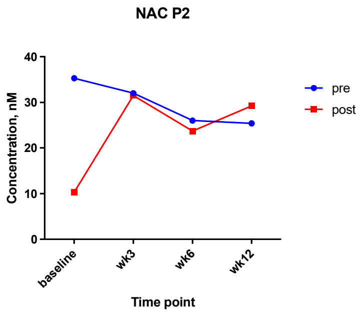
NAC P2
Concentration, nM
40
30
20
10
0
baseline
wk3
wk6
wk12
Time point
pre
post

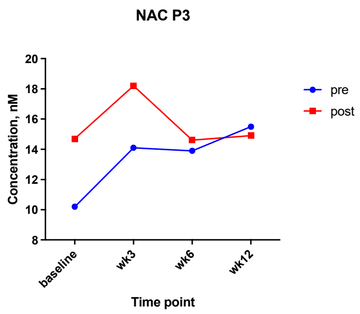
NAC P3
Concentration, nM
20
18
16
14
12
10
8
baseline
wk3
wk6
wk12
Time point
pre
post

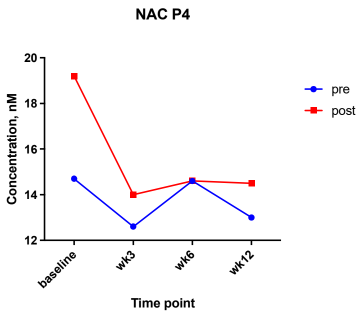
NAC P4
Concentration, nM
20
18
16
14
12
baseline
wk3
wk6
wk12
Time point
pre
post

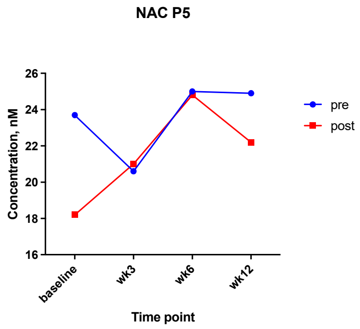
NAC P5
Concentration, nM
26
24
22
20
18
16
baseline
wk3
wk6
wk12
Time point
pre
post

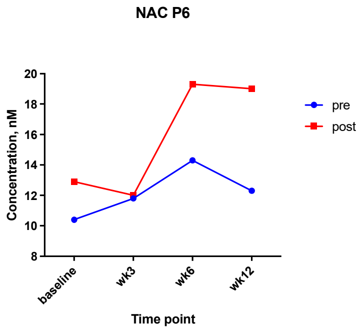
NAC P6
Concentration, nM
20
18
16
14
12
10
8
baseline
wk3
wk6
wk12
Time point
pre
post

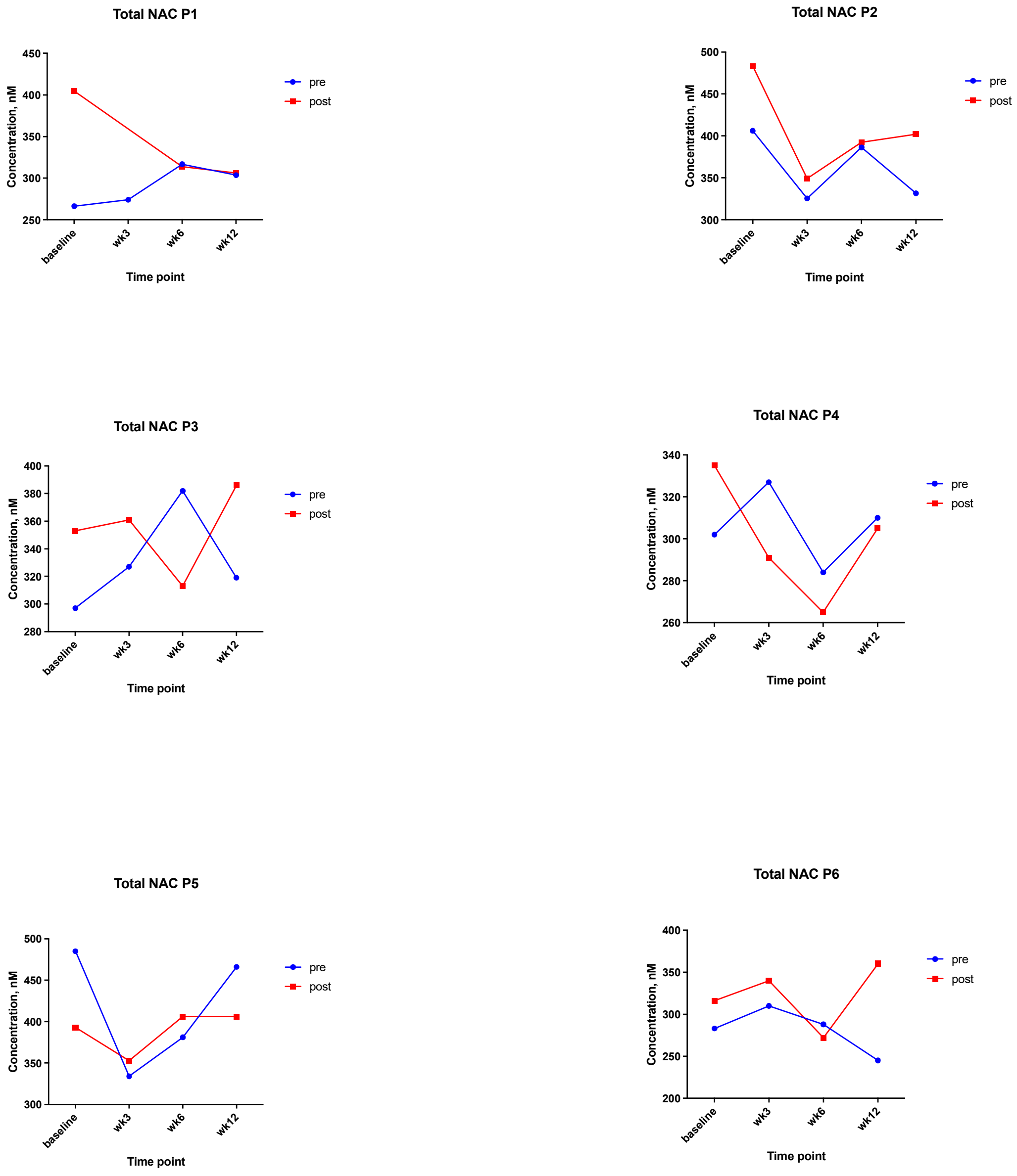
Total NAC P1
Concentration, nM
450
400
350
300
250
baseline
wk3
wk6
wk12
Time point
pre
post
Total NAC P2
Concentration, nM
500
450
400
350
300
baseline
wk3
wk6
wk12
Time point
pre
post
Total NAC P3
Concentration, nM
400
380
360
340
320
300
280
baseline
wk3
wk6
wk12
Time point
pre
post
Total NAC P4
Concentration, nM
340
320
300
280
260
baseline
wk3
wk6
wk12
Time point
pre
post
Total NAC P5
Concentration, nM
500
450
400
350
300
baseline
wk3
wk6
wk12
Time point
pre
post
Total NAC P6
Concentration, nM
400
350
300
250
200
baseline
wk3
wk6
wk12
Time point
pre
post

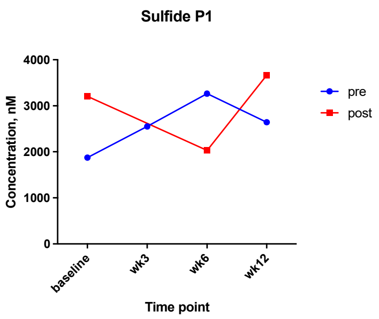
Sulfide P1
4000
3000
2000
1000
0
Concentration, nM
baseline
wk3
wk6
wk12
Time point
pre
post

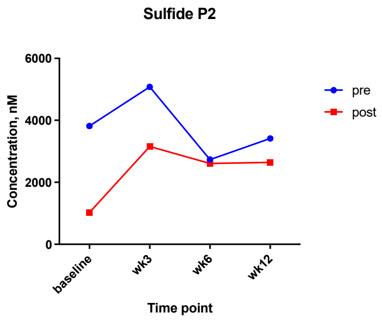
Sulfide P2
6000
4000
2000
0
Concentration, nM
baseline
wk3
wk6
wk12
Time point
pre
post

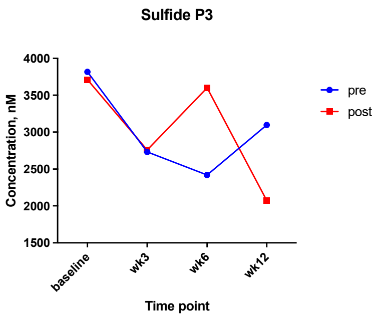
Sulfide P3
4000
3500
3000
2500
2000
1500
Concentration, nM
baseline
wk3
wk6
wk12
Time point
pre
post

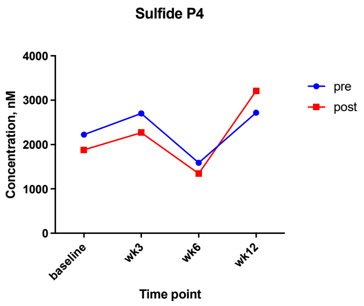
Sulfide P4
4000
3000
2000
1000
0
Concentration, nM
baseline
wk3
wk6
wk12
Time point
pre
post

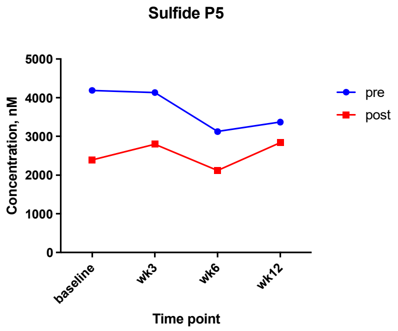
Sulfide P5
5000
4000
3000
2000
1000
0
Concentration, nM
baseline
wk3
wk6
wk12
Time point
pre
post

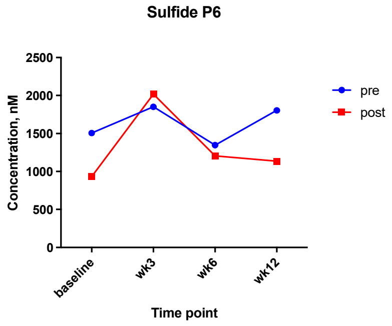
Sulfide P6
2500
2000
1500
1000
500
0
Concentration, nM
baseline
wk3
wk6
wk12
Time point
pre
post

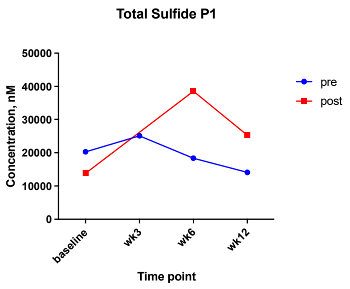
Total Sulfide P1
Concentration, nM
50000
40000
30000
20000
10000
0
baseline
wk3
wk6
wk12
Time point
pre
post

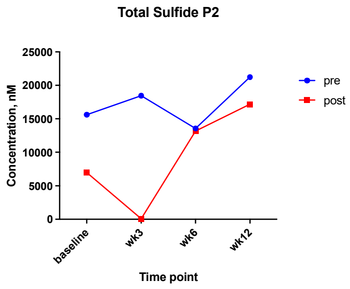
Total Sulfide P2
Concentration, nM
25000
20000
15000
10000
5000
0
baseline
wk3
wk6
wk12
Time point
pre
post

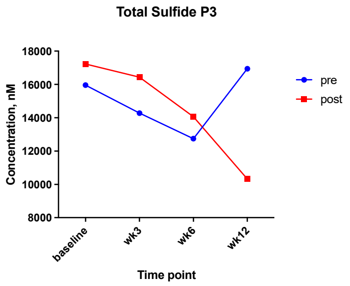
Total Sulfide P3
Concentration, nM
18000
16000
14000
12000
10000
8000
baseline
wk3
wk6
wk12
Time point
pre
post

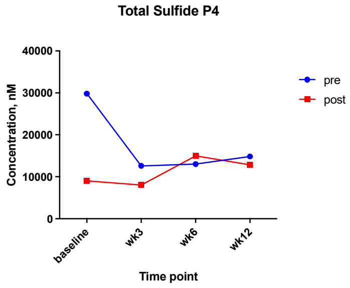
Total Sulfide P4
Concentration, nM
40000
30000
20000
10000
0
baseline
wk3
wk6
wk12
Time point
pre
post

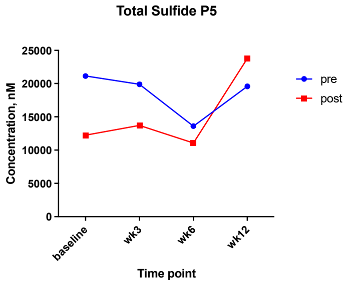
Total Sulfide P5
Concentration, nM
25000
20000
15000
10000
5000
0
baseline
wk3
wk6
wk12
Time point
pre
post

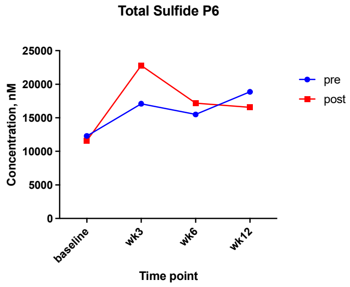
Total Sulfide P6
Concentration, nM
25000
20000
15000
10000
5000
0
baseline
wk3
wk6
wk12
Time point
pre
post

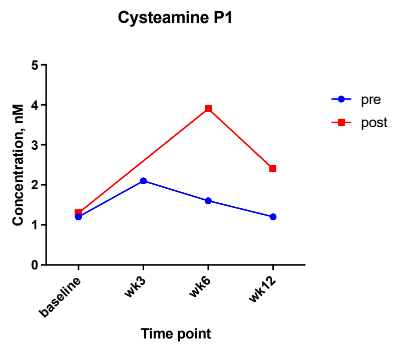
Cysteamine P1
Concentration, nM
0
1
2
3
4
5
baseline
wk3
wk6
wk12
Time point
pre
post

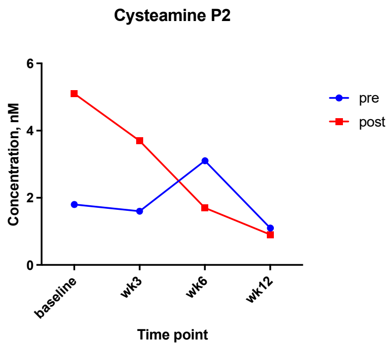
Cysteamine P2
Concentration, nM
0
2
4
6
baseline
wk3
wk6
wk12
Time point
pre
post

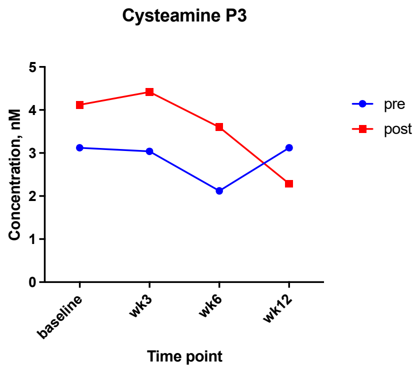
Cysteamine P3
Concentration, nM
0
1
2
3
4
5
baseline
wk3
wk6
wk12
Time point
pre
post

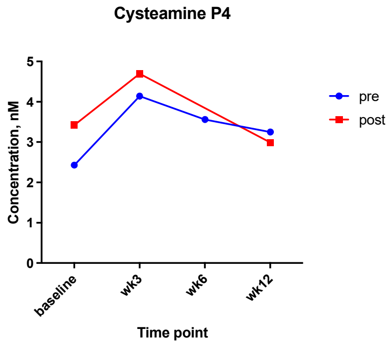
Cysteamine P4
Concentration, nM
0
1
2
3
4
5
baseline
wk3
wk6
wk12
Time point
pre
post

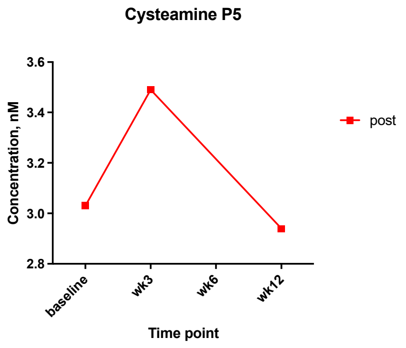
Cysteamine P5
Concentration, nM
2.8
3.0
3.2
3.4
3.6
baseline
wk3
wk6
wk12
Time point
post

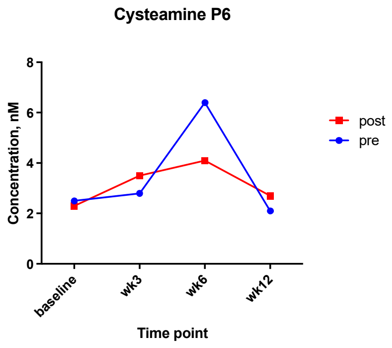
Cysteamine P6
Concentration, nM
0
2
4
6
8
baseline
wk3
wk6
wk12
Time point
post
pre

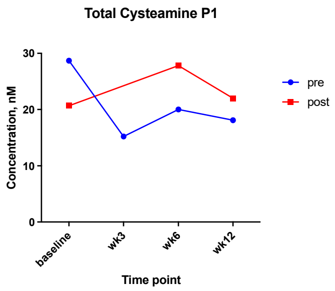
Total Cysteamine P1
Concentration, nM
0
10
20
30
baseline
wk3
wk6
wk12
Time point
pre
post

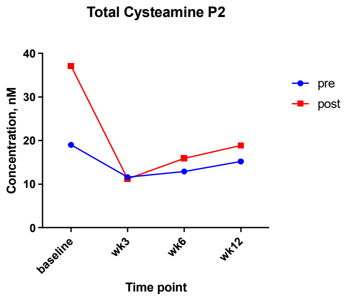
Total Cysteamine P2
Concentration, nM
0
10
20
30
40
baseline
wk3
wk6
wk12
Time point
pre
post

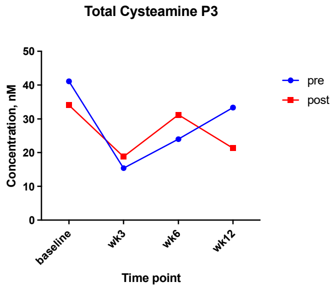
Total Cysteamine P3
Concentration, nM
0
10
20
30
40
50
baseline
wk3
wk6
wk12
Time point
pre
post

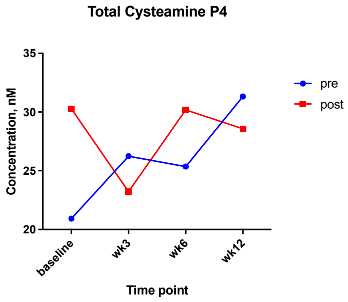
Total Cysteamine P4
Concentration, nM
20
25
30
35
baseline
wk3
wk6
wk12
Time point
pre
post

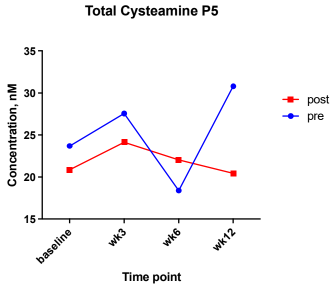
Total Cysteamine P5
Concentration, nM
15
20
25
30
35
baseline
wk3
wk6
wk12
Time point
post
pre

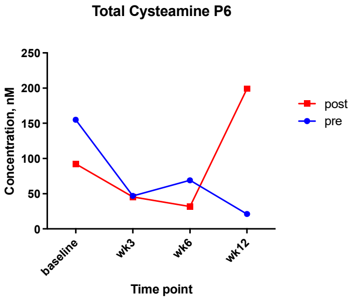
Total Cysteamine P6
Concentration, nM
0
50
100
150
200
250
baseline
wk3
wk6
wk12
Time point
post
pre

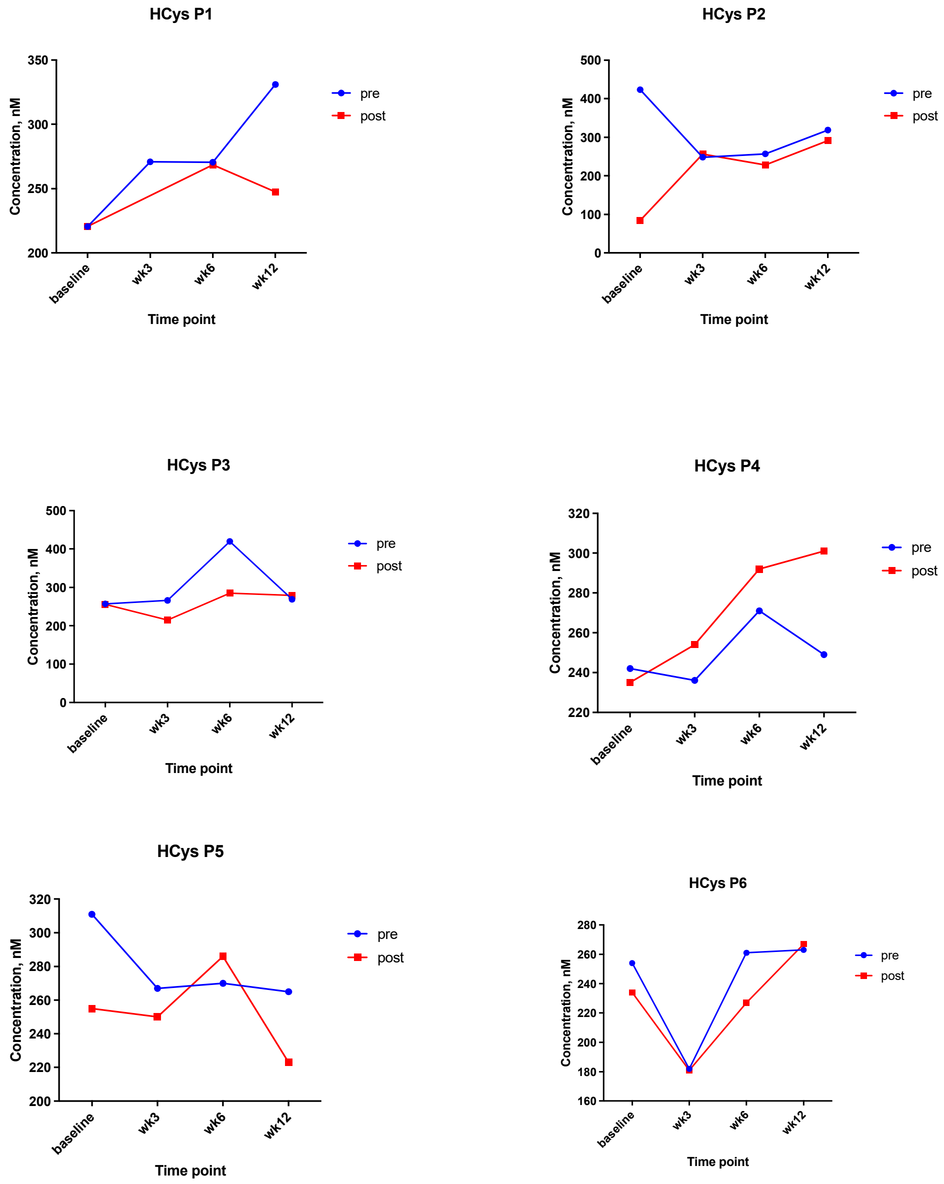
HCys P1
Concentration, nM
350
300
250
200
baseline
wk3
wk6
wk12
Time point
pre
post
HCys P2
Concentration, nM
500
400
300
200
100
0
baseline
wk3
wk6
wk12
Time point
pre
post
HCys P3
Concentration, nM
500
400
300
200
100
0
baseline
wk3
wk6
wk12
Time point
pre
post
HCys P4
Concentration, nM
320
300
280
260
240
220
baseline
wk3
wk6
wk12
Time point
pre
post
HCys P5
Concentration, nM
320
300
280
260
240
220
200
baseline
wk3
wk6
wk12
Time point
pre
post
HCys P6
Concentration, nM
280
260
240
220
200
180
160
baseline
wk3
wk6
wk12
Time point
pre
post

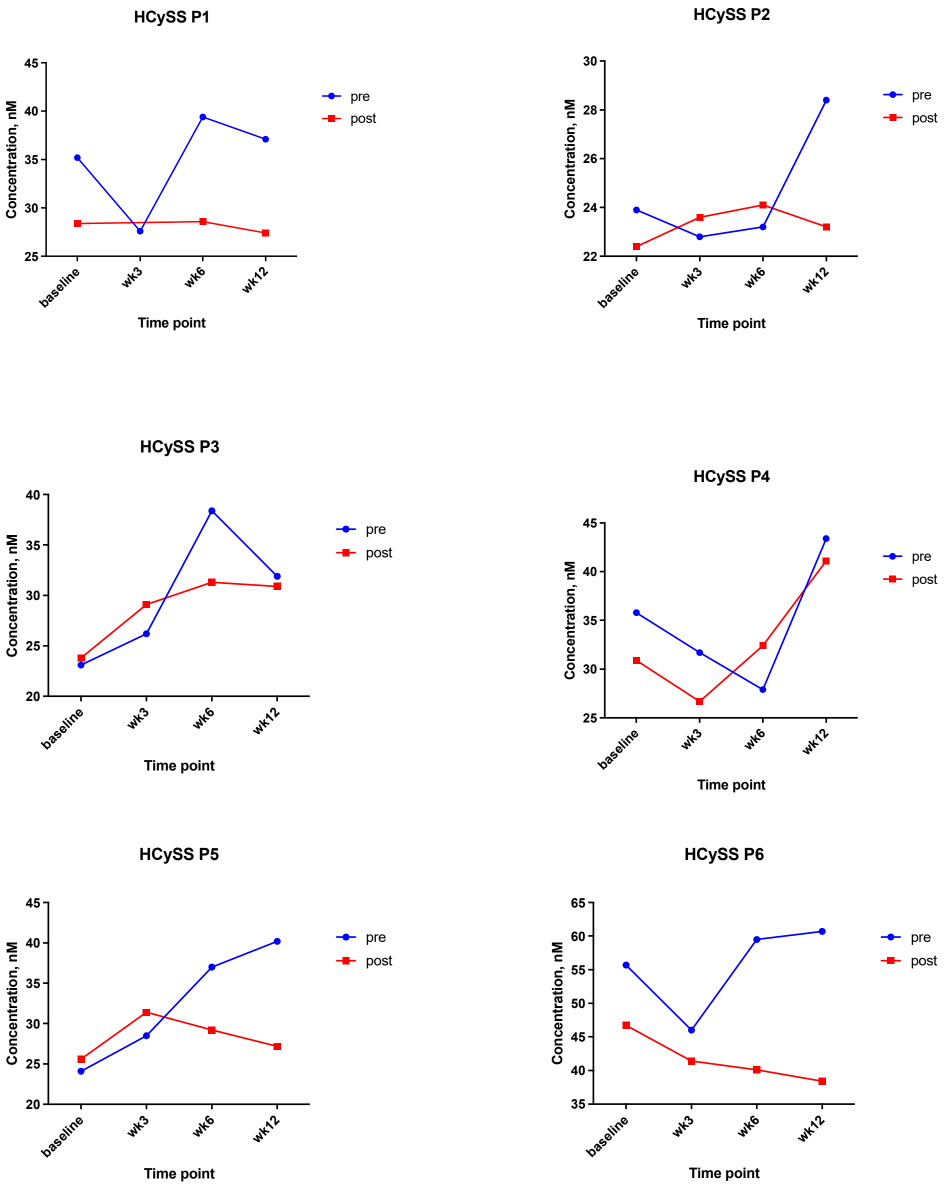
HCySS P1
Concentration, nM
45
40
35
30
25
baseline
wk3
wk6
wk12
Time point
pre
post
HCySS P2
Concentration, nM
30
28
26
24
22
baseline
wk3
wk6
wk12
Time point
pre
post
HCySS P3
Concentration, nM
40
35
30
25
20
baseline
wk3
wk6
wk12
Time point
pre
post
HCySS P4
Concentration, nM
45
40
35
30
25
baseline
wk3
wk6
wk12
Time point
pre
post
HCySS P5
Concentration, nM
45
40
35
30
25
20
baseline
wk3
wk6
wk12
Time point
pre
post
HCySS P6
Concentration, nM
65
60
55
50
45
40
35
baseline
wk3
wk6
wk12
Time point
pre
post

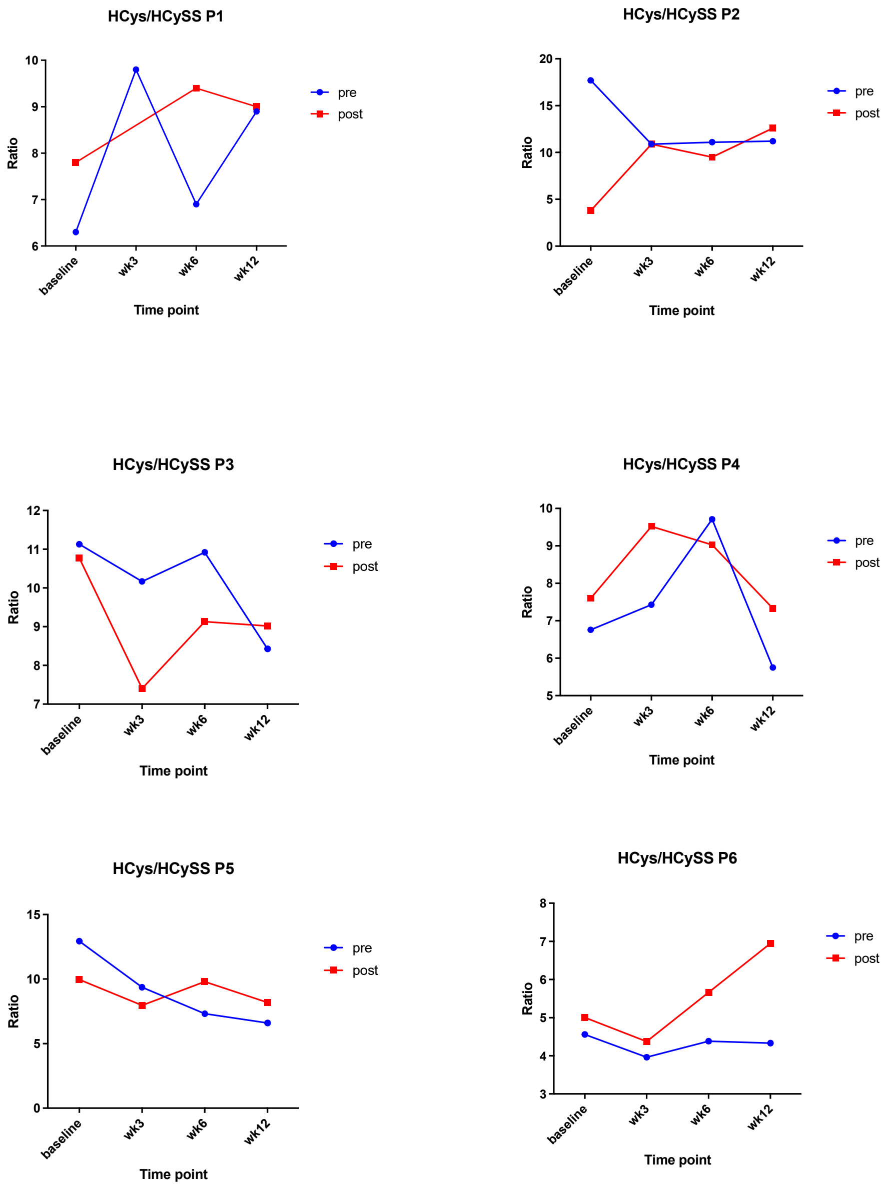
HCys/HCySS P1
HCys/HCySS P2
HCys/HCySS P3
HCys/HCySS P4
HCys/HCySS P5
HCys/HCySS P6
Ratio
Time point
baseline
wk3
wk6
wk12
pre
post

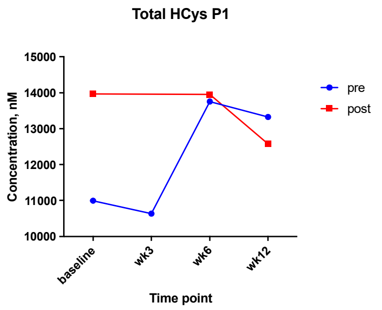
Total HCys P1
15000
14000
13000
12000
11000
10000
Concentration, nM
baseline
wk3
wk6
wk12
Time point
pre
post

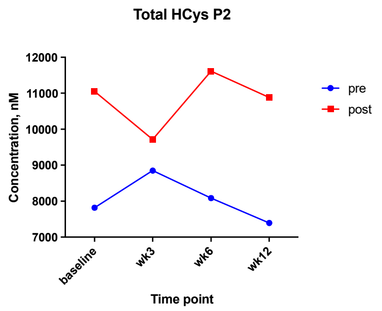
Total HCys P2
12000
11000
10000
9000
8000
7000
Concentration, nM
baseline
wk3
wk6
wk12
Time point
pre
post

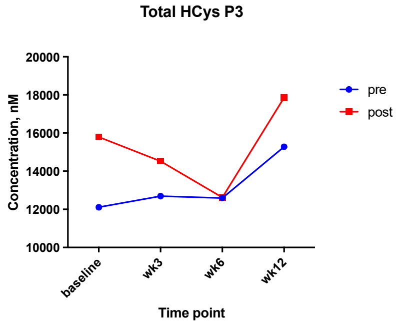
Total HCys P3
20000
18000
16000
14000
12000
10000
Concentration, nM
baseline
wk3
wk6
wk12
Time point
pre
post

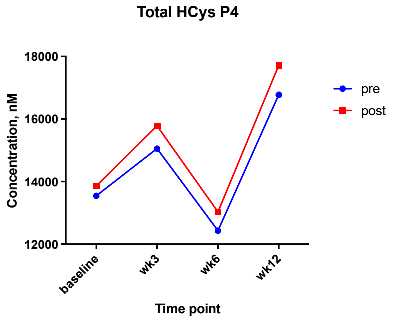
Total HCys P4
18000
16000
14000
12000
Concentration, nM
baseline
wk3
wk6
wk12
Time point
pre
post

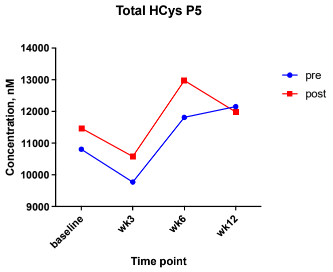
Total HCys P5
14000
13000
12000
11000
10000
9000
Concentration, nM
baseline
wk3
wk6
wk12
Time point
pre
post

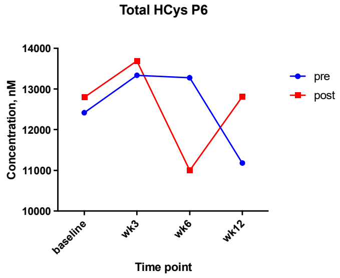
Total HCys P6
14000
13000
12000
11000
10000
Concentration, nM
baseline
wk3
wk6
wk12
Time point
pre
post

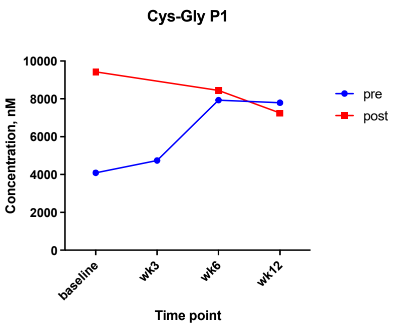
Cys-Gly P1
10000
8000
6000
4000
2000
0
Concentration, nM
baseline
wk3
wk6
wk12
Time point
pre
post

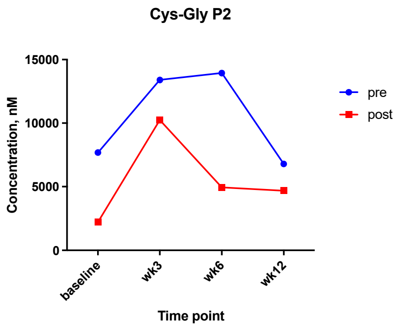
Cys-Gly P2
15000
10000
5000
0
Concentration, nM
baseline
wk3
wk6
wk12
Time point
pre
post

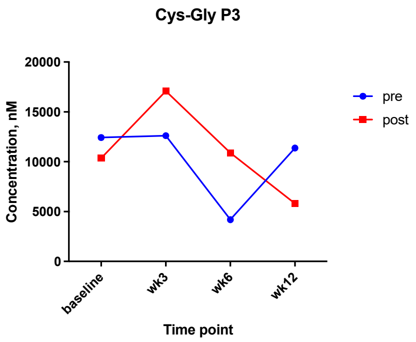
Cys-Gly P3
20000
15000
10000
5000
0
Concentration, nM
baseline
wk3
wk6
wk12
Time point
pre
post

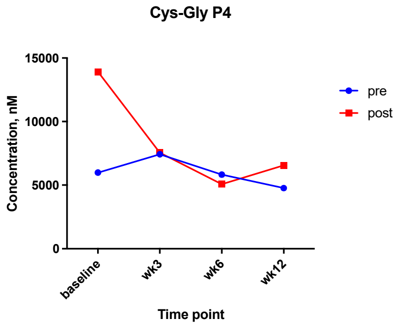
Cys-Gly P4
15000
10000
5000
0
Concentration, nM
baseline
wk3
wk6
wk12
Time point
pre
post

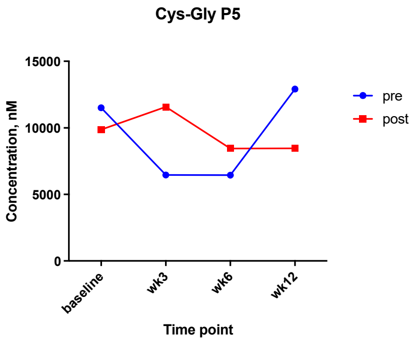
Cys-Gly P5
15000
10000
5000
0
Concentration, nM
baseline
wk3
wk6
wk12
Time point
pre
post

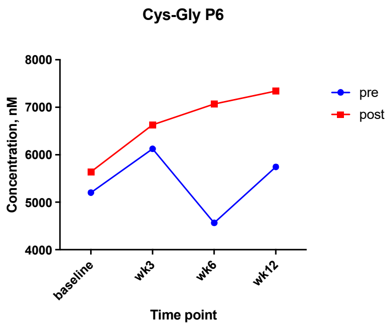
Cys-Gly P6
8000
7000
6000
5000
4000
Concentration, nM
baseline
wk3
wk6
wk12
Time point
pre
post

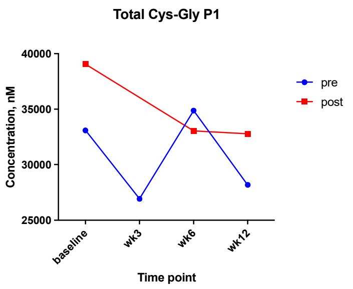
Total Cys-Gly P1
Concentration, nM
40000
35000
30000
25000
baseline
wk3
wk6
wk12
Time point
pre
post

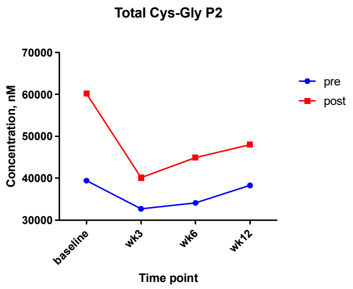
Total Cys-Gly P2
Concentration, nM
70000
60000
50000
40000
30000
baseline
wk3
wk6
wk12
Time point
pre
post

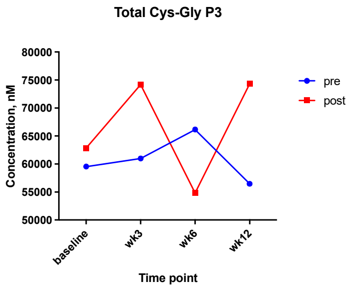
Total Cys-Gly P3
Concentration, nM
80000
75000
70000
65000
60000
55000
50000
baseline
wk3
wk6
wk12
Time point
pre
post

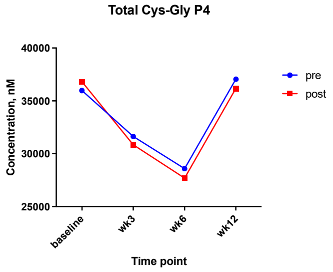
Total Cys-Gly P4
Concentration, nM
40000
35000
30000
25000
baseline
wk3
wk6
wk12
Time point
pre
post

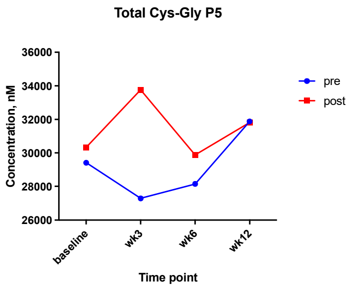
Total Cys-Gly P5
Concentration, nM
36000
34000
32000
30000
28000
26000
baseline
wk3
wk6
wk12
Time point
pre
post

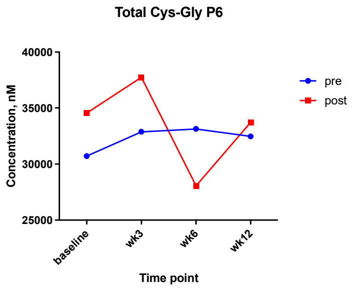
Total Cys-Gly P6
Concentration, nM
40000
35000
30000
25000
baseline
wk3
wk6
wk12
Time point
pre
post

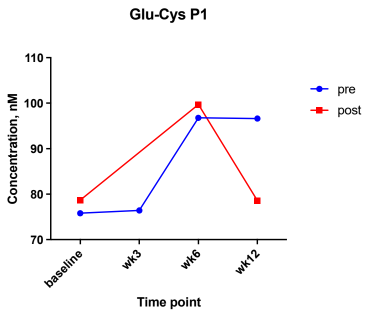
Glu-Cys P1
Concentration, nM
110
100
90
80
70
pre
post
baseline
wk3
wk6
wk12
Time point

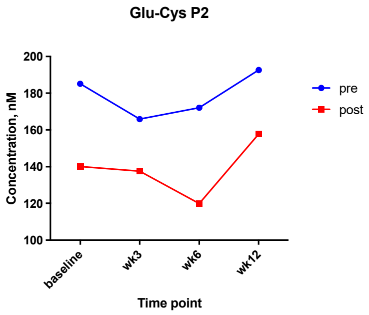
Glu-Cys P2
Concentration, nM
200
180
160
140
120
100
pre
post
baseline
wk3
wk6
wk12
Time point

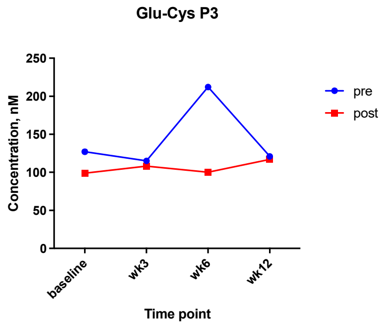
Glu-Cys P3
Concentration, nM
250
200
150
100
50
0
pre
post
baseline
wk3
wk6
wk12
Time point

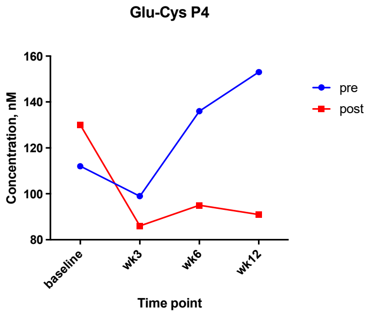
Glu-Cys P4
Concentration, nM
160
140
120
100
80
pre
post
baseline
wk3
wk6
wk12
Time point

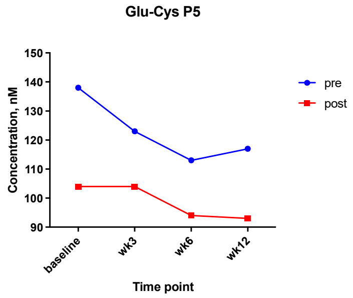
Glu-Cys P5
Concentration, nM
150
140
130
120
110
100
90
pre
post
baseline
wk3
wk6
wk12
Time point

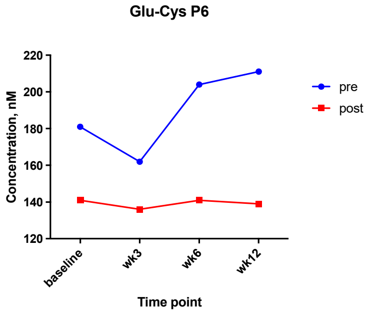
Glu-Cys P6
Concentration, nM
220
200
180
160
140
120
pre
post
baseline
wk3
wk6
wk12
Time point

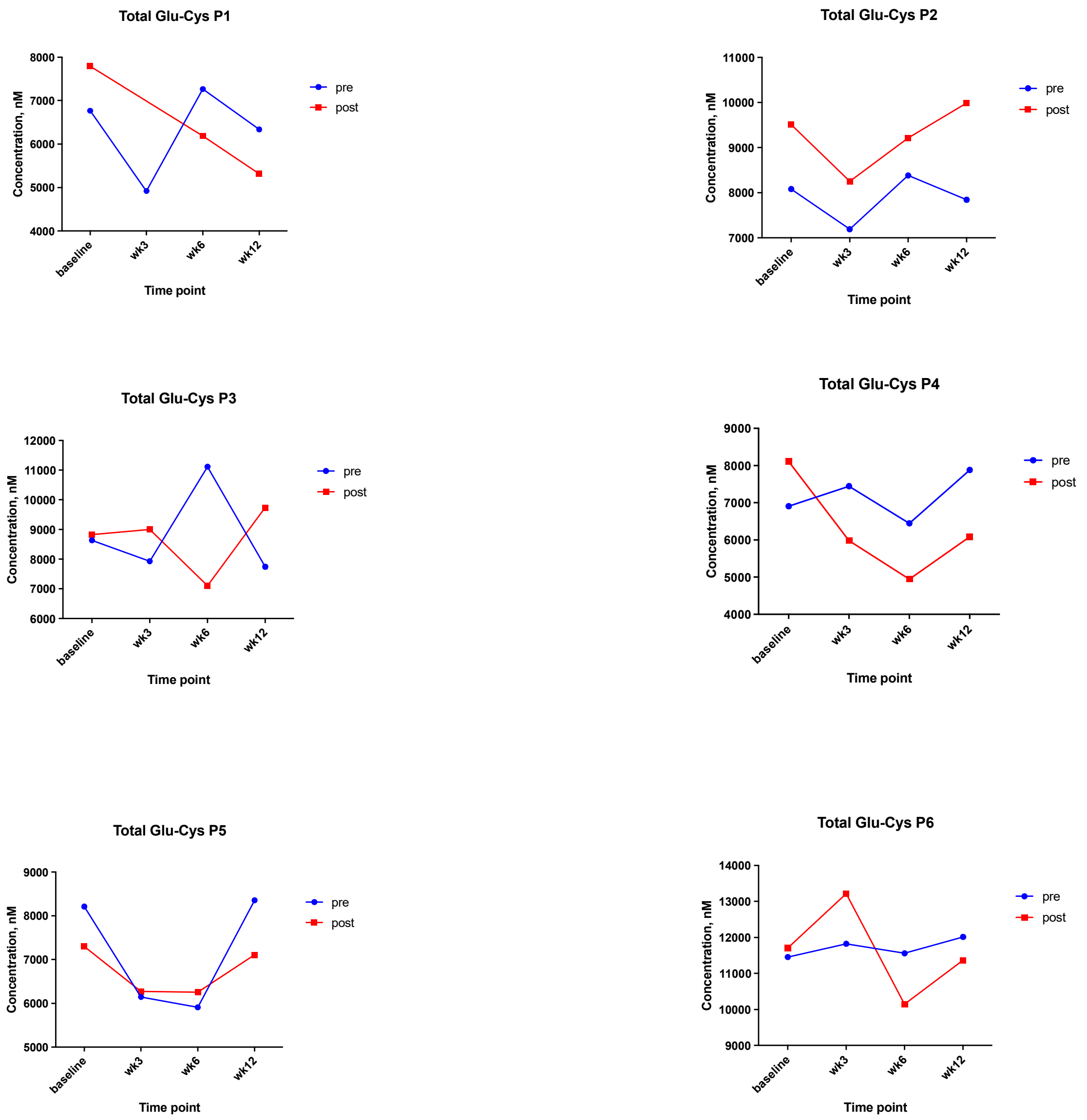

Figure S4 Individual responses of the free and total thiol metabolome to exercise. Data presented for individual patients (n=6). Red line = pre acute exercise challenge, blue line = post acute exercise challenge. Abbreviations GSH: reduced gluthathione; GSSG: oxidised glutathione; cys:cysteine, cyss: cystine, hcys: homocysteine, hcyss:homocystine NAC; N-acetylcysteine, Cys-Gly; cysteinylglycine; Glu-Cys; L-Glutamyl-L-Cysteine

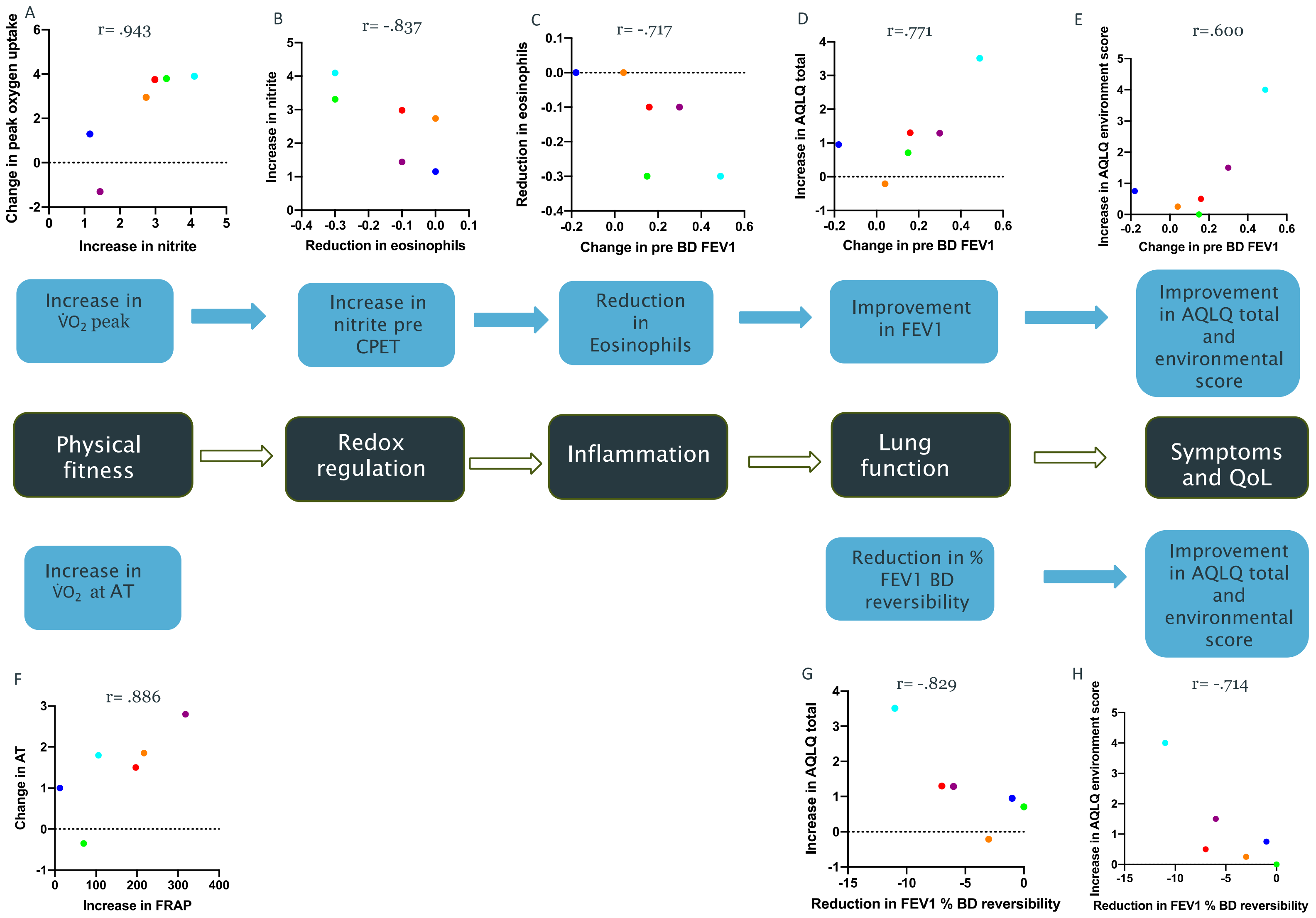
A
r= .943
Change in peak oxygen uptake
Increase in nitrite
B
r= -.837
Increase in nitrite
Reduction in eosinophils
C
r= -.717
Reduction in eosinophils
Change in pre BD FEV1
D
r=.771
Increase in AQLQ total
Change in pre BD FEV1
E
r=.600
Increase in AQLQ environment score
Change in pre BD FEV1
Increase in V̇O2 peak
Increase in nitrite pre CPET
Reduction in Eosinophils
Improvement in FEV1
Improvement in AQLQ total and environmental score
Physical fitness
Redox regulation
Inflammation
Lung function
Symptoms and QoL
Increase in V̇O2 at AT
Reduction in % FEV1 BD reversibility
Improvement in AQLQ total and environmental score
F
r= .886
Change in AT
Increase in FRAP
G
r= -.829
Increase in AQLQ total
Reduction in FEV1 % BD reversibility
H
r= -.714
Increase in AQLQ environment score
Reduction in FEV1 % BD reversibility

Figure S5._Significant associations (Spearman's Test) between physical fitness and clinical asthma symptoms. On the top row, A) a greater increase in maximum oxygen uptake is associated with a greater increase in pre CPET nitrite from baseline (r=.943). B) A greater increase in pre CPET nitrite is significantly associated with a greater the reduction in eosinophil levels (r=-.837). C) A greater reduction in eosinophils is associated with a greater increase in pre-BD FEV1 (r=-717), and a D) greater increase in FEV1 correlates with a greater increase in AQLQ total score (r=.771) and E) AQLQ environmental score (r=.600). On the bottom row, F) a greater increase in oxygen uptake at AT is associated with a larger increase in FRAP. G and H) A greater reduction in FEV1% BD reversibility negatively correlates with a greater increase in AQLQ total and environmental scores (r=-.829 and -.714). To link the two rows, a greater reduction in airways hyper-reactivity as assessed by reduction in %FEV1 BD reversibility negatively correlates with a greater increase in FEV1 in litres (r=-.771; data not shown) Abbreviations: AT; anaerobic threshold, CPET; cardiopulmonary exercise test; BD; bronchodilator, FEV1; forced expiratory volume in 1 second, FRAP; ferric reducing antioxidant capacity of plasma. An r value of > 0.7 is considered a strong correlation, 0.4-0.7 a moderate correlation.

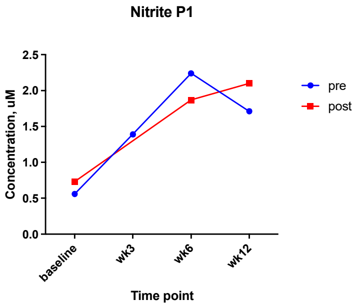
Nitrite P1
2.5
2.0
1.5
1.0
0.5
0.0
Concentration, uM
baseline
wk3
wk6
wk12
Time point
pre
post

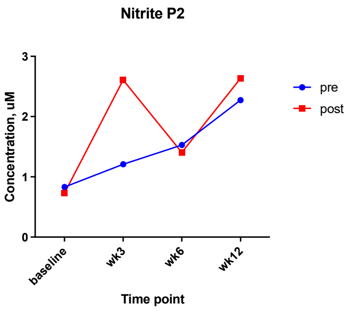
Nitrite P2
3
2
1
0
Concentration, uM
baseline
wk3
wk6
wk12
Time point
pre
post

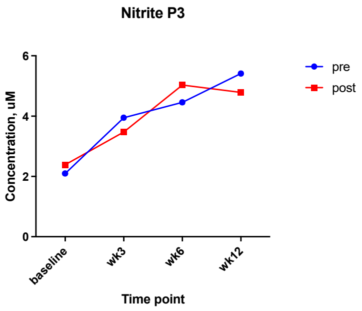
Nitrite P3
6
4
2
0
Concentration, uM
baseline
wk3
wk6
wk12
Time point
pre
post

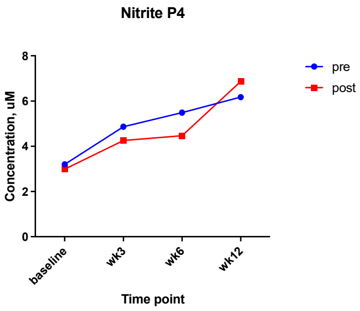
Nitrite P4
8
6
4
2
0
Concentration, uM
baseline
wk3
wk6
wk12
Time point
pre
post

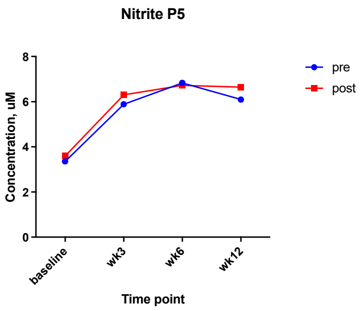
Nitrite P5
8
6
4
2
0
Concentration, uM
baseline
wk3
wk6
wk12
Time point
pre
post

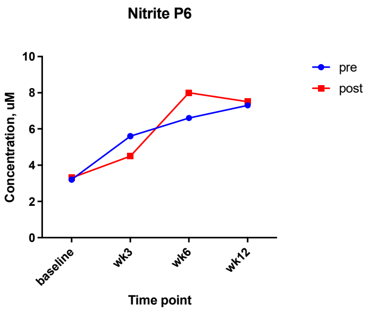
Nitrite P6
10
8
6
4
2
0
Concentration, uM
baseline
wk3
wk6
wk12
Time point
pre
post

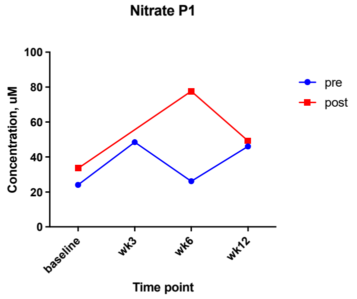
Nitrate P1
Concentration, uM
100
80
60
40
20
0
baseline
wk3
wk6
wk12
Time point
pre
post

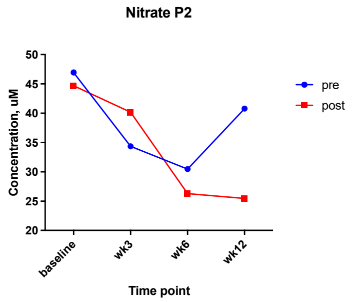
Nitrate P2
Concentration, uM
50
45
40
35
30
25
20
baseline
wk3
wk6
wk12
Time point
pre
post

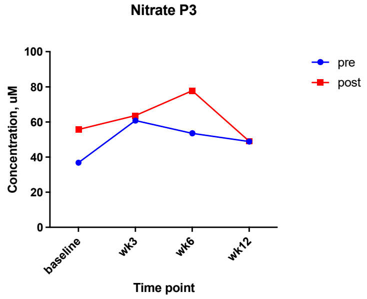
Nitrate P3
Concentration, uM
100
80
60
40
20
0
baseline
wk3
wk6
wk12
Time point
pre
post

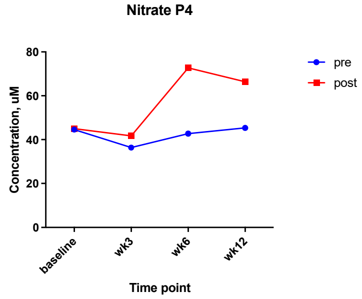
Nitrate P4
Concentration, uM
80
60
40
20
0
baseline
wk3
wk6
wk12
Time point
pre
post

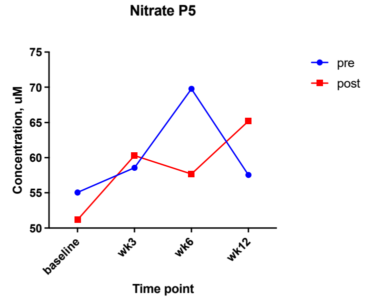
Nitrate P5
Concentration, uM
75
70
65
60
55
50
baseline
wk3
wk6
wk12
Time point
pre
post

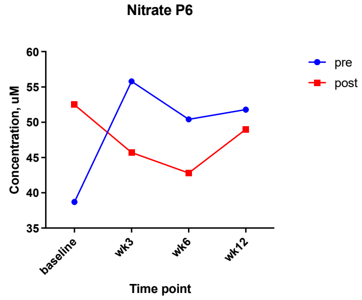
Nitrate P6
Concentration, uM
60
55
50
45
40
35
baseline
wk3
wk6
wk12
Time point
pre
post

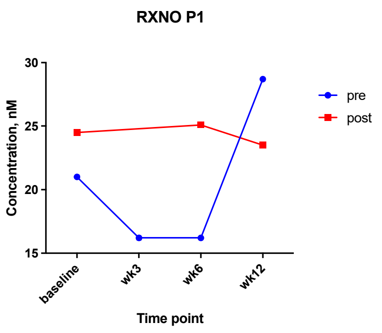
RXNO P1
Concentration, nM
30
25
20
15
baseline
wk3
wk6
wk12
Time point
pre
post

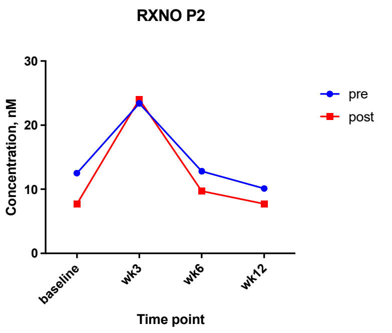
RXNO P2
Concentration, nM
30
20
10
0
baseline
wk3
wk6
wk12
Time point
pre
post

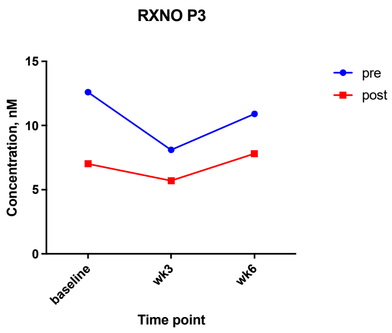
RXNO P3
Concentration, nM
15
10
5
0
baseline
wk3
wk6
Time point
pre
post

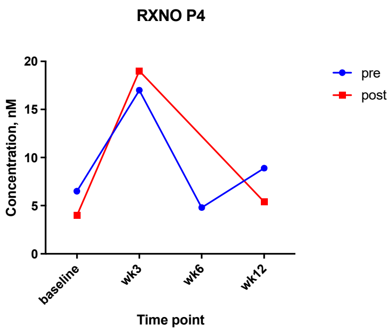
RXNO P4
Concentration, nM
20
15
10
5
0
baseline
wk3
wk6
wk12
Time point
pre
post

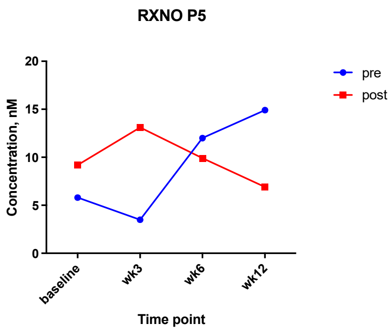
RXNO P5
Concentration, nM
20
15
10
5
0
baseline
wk3
wk6
wk12
Time point
pre
post

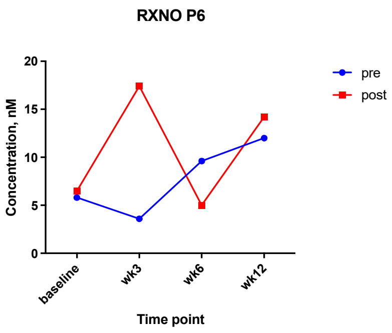
RXNO P6
Concentration, nM
20
15
10
5
0
baseline
wk3
wk6
wk12
Time point
pre
post

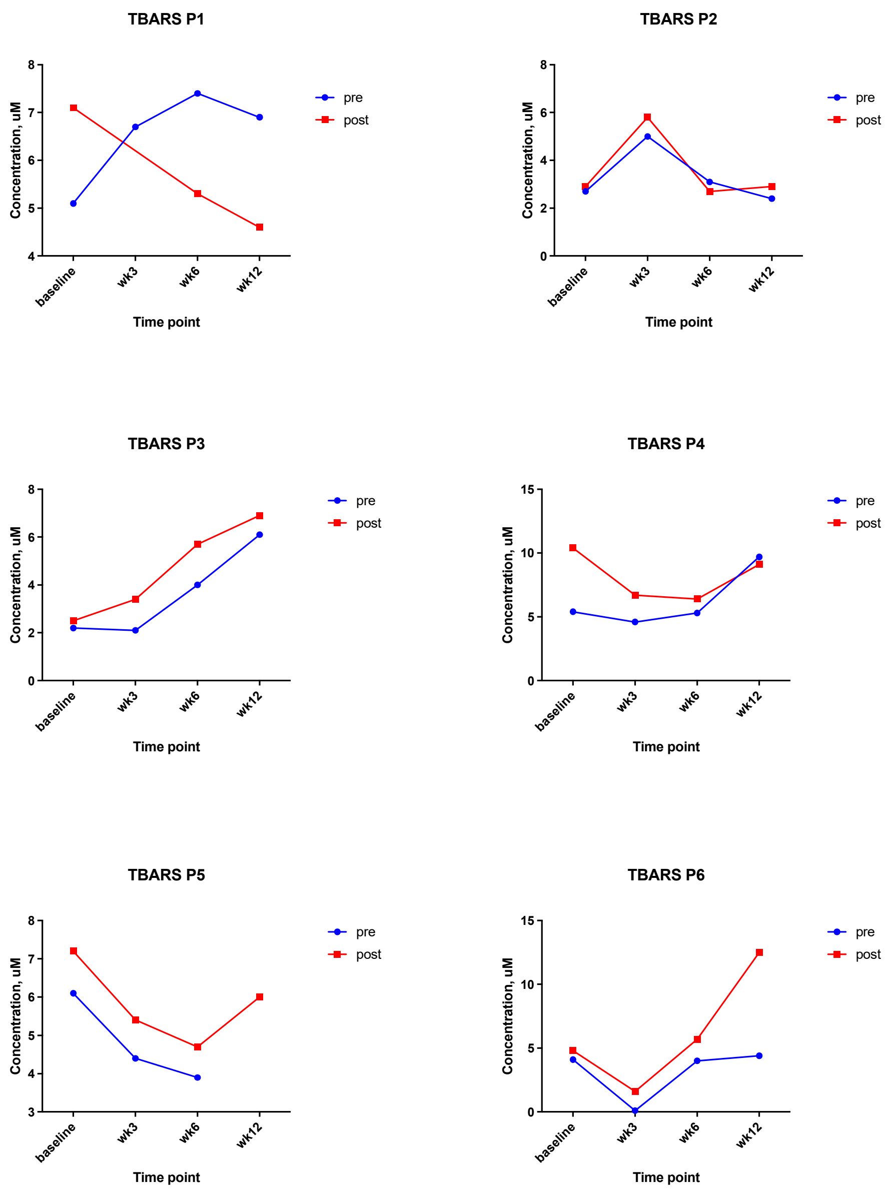
TBARS P1
Concentration, uM
Time point
baseline
wk3
wk6
wk12
pre
post
TBARS P2
Concentration, uM
Time point
baseline
wk3
wk6
wk12
pre
post
TBARS P3
Concentration, uM
Time point
baseline
wk3
wk6
wk12
pre
post
TBARS P4
Concentration, uM
Time point
baseline
wk3
wk6
wk12
pre
post
TBARS P5
Concentration, uM
Time point
baseline
wk3
wk6
wk12
pre
post
TBARS P6
Concentration, uM
Time point
baseline
wk3
wk6
wk12
pre
post

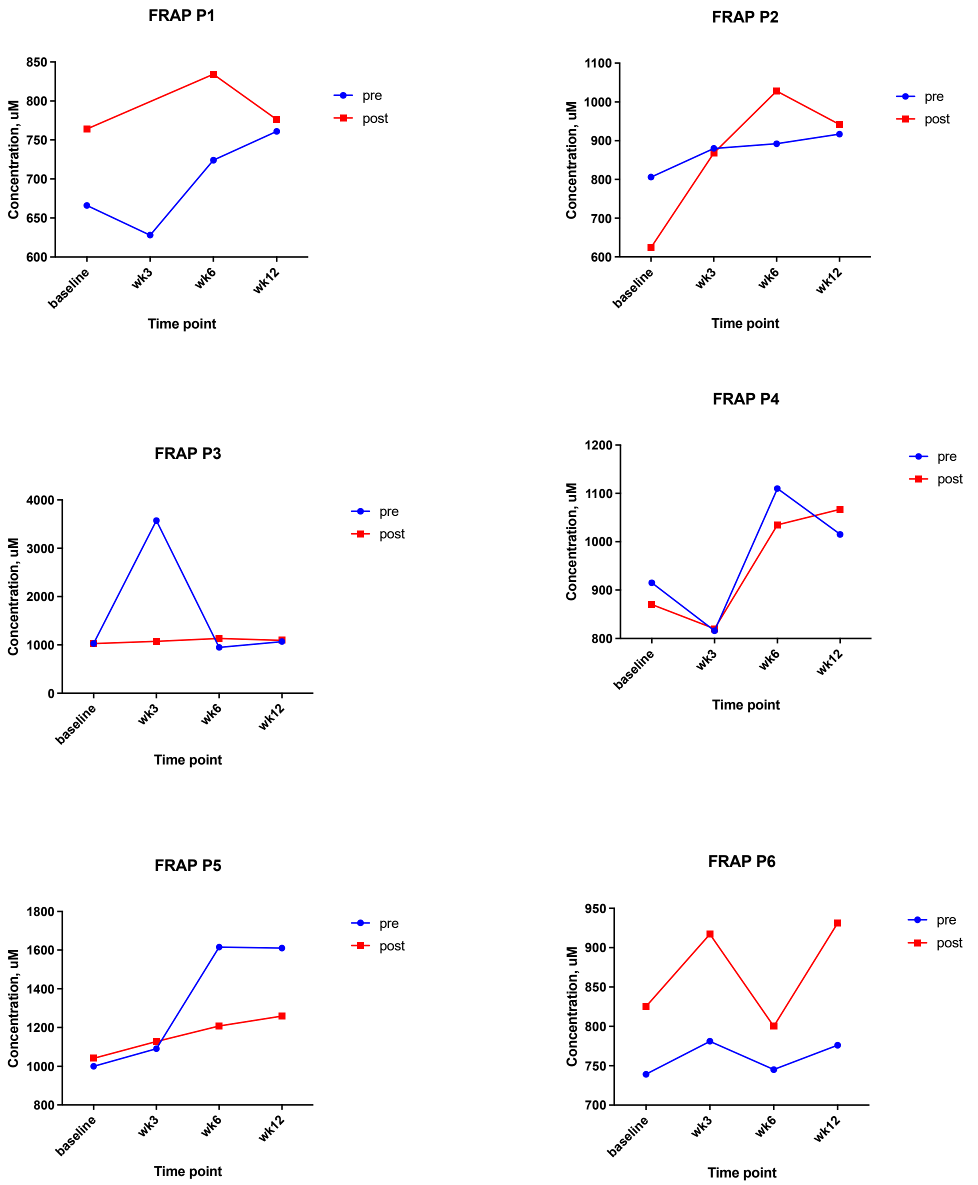

Figure S6 Individual responses of nitrospecies, TBARS and FRAP to exercise. Data presented for individual patients (n=6). Red line = pre acute exercise challenge, blue line = post acute exercise challenge. Abbreviations; RXNO; other nitrosospecies; TBARS; thiobarbituric acid reactive substances, FRAP; Ferric reducing antioxidant capacity of plasma
